# MIMO Volterra kernel recovery in the frequency domain using neural networks

**DOI:** 10.1007/s11071-026-12672-9

**Published:** 2026-07-03

**Authors:** O. H. L. Preston, T. J. Rogers, K. Worden

**Affiliations:** Dynamics Research Group, School of Mechanical, Aerospace and Civil Engineering, University of Sheffield, Mappin Street, Sheffield, S1 3JD UK

**Keywords:** Volterra series, Neural networks, Higher-order frequency response functions, Multi-degree-of-freedom systems

## Abstract

This paper presents a detailed analysis of a process for recovering multi-degree-of-freedom Volterra kernels using neural network weights, addressing the fact that such Higher-order Frequency Response Functions (HFRFs) have not previously been directly recovered from data. A novel method is proposed for HFRF recovery of Volterra kernels up to third order, and its effectiveness is demonstrated on a variety of systems using simulated data for validation. The harmonic-probing algorithms are derived from a general multi-input-multi-output NARX neural network model. These algorithms recover Volterra kernels for time-invariant systems with up to *n* degrees of freedom. The results demonstrate the accuracy of the method and suggest a promising direction for HFRF recovery in nonlinear time-invariant systems.

## Introduction

Working with nonlinear dynamical systems with multiple degrees of freedom is common in most engineering systems applications. The common methods for modelling such systems often rely on numerically solving high-dimensional differential equations which can be very challenging, and difficult to interpret. It has been shown that the Volterra series can be a powerful technique to generalise linear convolution theory to nonlinear systems. The Volterra series method of recovery is completed by representing the output as multiple different convolutions like a Taylor series of functionals [1]. The resulting kernels provide insight into the underlying mechanisms of the system, enabling an analysis of not only the linear dynamics but also the higher-order kernels that characterize quadratic, cubic, and other higher-order contributions. So far, the single-input single-output (SISO) Volterra series model has gained the most attention because of its relative simplicity [2–4]. As the Volterra series order increases, the terms required to determine such kernels grows; therefore less effort has been focussed on solving multi-input-multi-output (MIMO) models, which greatly limits the information that can be retrieved to solve real-world problems. With important simplifications, suitable machine-learning methods, and modern improvements in data processing, it is now possible to use MIMO Volterra series techniques to understand complex systems.

Adopting theoretical work in multi-degree-of-freedom (MDOF) system Volterra kernel recovery [4, 5], one can take advantage of the symmetries in the kernels to develop much simpler representations for the relationships between the kernels higher-order frequency response functions (HFRF) and the measured outputs. It can be shown that this theory can then simply be extended, as in the work of Chance et al. [3], by adapting the neural-network (NN) architecture in that work to accommodate the growing kernel relationships.

Improvements in computational efficiency have led to much more consistent predictions for the Volterra kernels of unknown time-invariant systems using different machine-learning architectures [6, 7]. Therefore, it is necessary to extend the nonlinear autoregressive with exogenous inputs

(NARX) neural network by accommodating the MDOF theory to analyse nonlinear MIMO systems with unknown underlying nonlinearity, where the neural network can help to identify it. Since neural-network architectures are both popular and powerful, introducing interpretability to a larger set of systems provides potential for further forecasting improvements and provides an opportunity for further machine-learning methods to accommodate this development.

The objective of this paper is to extend the SISO Volterra series neural network approach to MIMO systems, easing the burden of calculating the complicated cross-kernels and promoting the potential for interpretability of more complex system dynamics. The layout of this paper is as follows: Sect. 2 discusses the theory behind recovering the MIMO Volterra kernels and how this can be adapted to the neural-network structure. Section 3 then proceeds to illustrate the application of the theory to simple nonlinear spring-mass-damper systems and how the theory can be adapted to *n*-DOF systems in both continuous-time, (based on the equations of motion) and discrete-time, (in terms of the neural network weights using a NARX approach). Section 4 then presents the results of training such networks on data from 2DOF and 5DOF systems with varying noise, validating theory.

### Related work

The Volterra series approach to understanding nonlinear systems has been used for a variety of scenarios; these range from manipulating machine-learning architectures to accommodate its structure, to improving identification of different levels of nonlinearity in theoretical and real-world applications. The Volterra series assists in separating linear and nonlinear response contributions, helping to interpret the nonlinearity via kernels. Allowing differentiation between nonlinearity and system damage, amongst many other benefits, making it an excellent and unique tool, as will be shown.

Observing examples of theoretical approaches to MDOF systems analysis, the Volterra series has been used alongside Gaussian Processes (GPs) to accommodate both single and multi-output systems in the time-domain using the process convolution formalism [8]; in this case, the kernels representing the nonlinearity are not observable, therefore the approach misses out on recovering valuable system information regarding the nonlinearity. GPs have recently been used to learn the Volterra kernels nonparametrically for multi-output systems [9] also in the time domain, but this doesn’t consider multiple-input contributions and therefore higher-order kernel estimates availability is limited. Many interesting multi-output Volterra models have been generated, such as the work of Scussel et al. [10] which provides a theoretical extension to the harmonic probing algorithm to observe the kernels, where only the output response is available using a Kautz filter. This, also limits the strength of kernel interpretability without input data available but would be very useful in systems where input data is unavailable such as large-scale structures like bridges or buildings. Interestingly a different method of approximating the HFRFs of output-only systems was implemented by Peng [11] to evaluate nonlinear contributions and understand crack locations in beams. However, neither method is applicable for understanding MIMO systems, where a framework would be very desirable to interpret nonlinear systems.

As has been highlighted, the Volterra series is effective in recovering nonlinear contributions to outputs or understanding the levels of nonlinearity by inspecting higher-order kernels. The theory is therefore very useful to implement for different nonlinear systems. It has been well adopted recently in mechanical, aerospace and civil systems, such as work in reduced-order modelling of unsteady transonic aerodynamic loads [12] using least-squares. This method uses small input loads to recover linear contributions and then increases the level of forcing to separate the nonlinearity via convolutions. Aeroelastic linear and quadratic nonlinear responses to sweep excitations [13] have also been recovered using the Volterra series. These, along with a variety of least-squares-based nonlinear kernel recovery or output recovery are quite common [14–16] but are limited by the level of nonlinearity and the information that can be gathered from them. In addition to this, machine-learning approaches have been applied to transonic aerodynamics via GPs and NNs to recover kernel coefficients linked to linear and quadratic contributions [17] or in a MIMO case, by altering the structure of a deep neural network to behave as the Volterra series to recover tidal responses [18], giving a means of MIMO Volterra kernel recovery. So far however, these architectures have only been applied to impose the Volterra series framework on the input–output relationship, but this does present a desirable opportunity to recover more information about the system.

The current research is mainly focused on time-domain results, by either recovering the possible linear output of a nonlinear system or nonlinear kernels, which are limited to low levels of nonlinearity and prone to overfitting. When input forcing measurements are accompanied by output data, such as in modern NARX methods like GP-NARX on physical systems [7, 19], one can operate in the frequency domain via harmonic probing of NARX based equations; producing clean, interpretable levels of nonlinearity. The harmonic probing approach, using NARX frameworks to recover HFRFs, allows more nonlinear interpretability than just the quadratic contribution, helping to understand systems better. Exceptions such as recent work from Schoukens et al. [20, 21], uses regularisation to estimate the nonlinear systems kernels, but this specific approach is limited to recovering time-domain kernels. Earlier work using RKHS by Dodd and Harrison [22, 23] also uses a regularised LS approach, but to instead estimate HFRFs; however, the reference [23] (although accommodating infinite degree Volterra series) only shows a comparison with ground truth for $H_1$. Interesting work by Butlin et al. [24–26] use GPs to recover orthogonal ‘Wiener kernels’. However, these kernels hold different properties and cannot be recovered by probing NN’s. So far, when considering MIMO systems, the NARX approach has only been implemented theoretically [4], therefore applying the harmonic-probing algorithm with MIMO kernel relationships to machine-learning algorithms help to observe the nonlinear interactions. As a result of its effectiveness in modelling abstract MIMO nonlinear interactions, probing simplicity [3] and ability to process an abundance of data, the NN was an obvious choice to recover the MIMO HFRFs with. The research therefore needed to achieve an efficient and versatile neural network related method of recovering MIMO Volterra kernels for MIMO systems adaptable to accommodate the available input–output data channels.

## MIMO Volterra series

It has been shown that for linear time-invariant systems

(LTIs), using Duhamel’s integral to convolve inputs in the time domain with a kernel ‘$\textit{h}_1$’ forecasts the output of the system ‘$y_1$’. The properties of the system are therefore encoded within ‘$\textit{h}_1$’, where its form can be derived by decomposing the known equation of motion for the system as [27],

1 $$\begin{aligned} y_1(t) = \int _{0}^{\infty }h_1(\tau )x(t-\tau )d\tau . \end{aligned}$$

The Fourier transform of equation (1) leads to the desirable form (2),

2 $$\begin{aligned} Y_1(\omega ) = H_1(\omega )X(\omega ), \end{aligned}$$

indicating that convolution in the time domain corresponds to multiplication in the frequency domain.

As this paper is focussed on the application of multiple degrees of freedom, it follows simply from superposition that the linear component of the output at a measured location is governed by the relationship between the other degrees of freedom inputs and relevant kernels,

3 $$\begin{aligned} \begin{aligned} y_1^{(p)}(t)&= \int _{0}^{\infty } h_1^{(p:1)}(\tau ) x^{(1)}(t - \tau ) d\tau \\&+ \int _{0}^{\infty } h_1^{(p:2)}(\tau ) x^{(2)}(t - \tau ) d\tau + \cdots \\&+ \int _{0}^{\infty } h_1^{(p:n)}(\tau ) x^{(n)}(t - \tau ) d\tau . \end{aligned} \end{aligned}$$

Therefore, for a system of *n*-DOFs as shown in Eq. (3), the output at point *p* is made up of the contribution of many kernels ‘*h*’. Each kernel represents a relationship between the output at point *p* and input contribution of each degree-of-freedom up to *n*.

As with the single-degree-of-freedom (SDOF) case in Eqs. (1) and (2); if there exists a nonlinear relationship between the input and output of the system, the convolution approach can be extended by forming a generalisation of the Taylor series using functionals commonly named the Volterra series [1]. Intuitively, $y_1$ is the linear contribution, $y_2$ the quadratic and so on. Therefore, a system can be comprised of many orders, up to the point that the specific output contribution is negligible to the overall output (in this case *k*),

4 $$\begin{aligned} y(t) = y_1(t) + y_2(t) + \cdots + y_k(t), \end{aligned}$$

where,

5 $$\begin{aligned} y_2(t) = \int _{0}^{\infty }\int _{0}^{\infty }h_2(\tau _1,\tau _2)x(t-\tau _1)x(t-\tau _2)d\tau _1d\tau _2 \end{aligned}$$

and generally,

6 $$\begin{aligned} \begin{aligned} y_k(t)&= \int _{0}^{\infty }\cdots \int _{0}^{\infty }h_k(\tau _1,\ldots ,\tau _k)x(t-\tau _1)\\&x(t-\tau _2) \cdots x(t-\tau _k)d\tau _1d\tau _2\cdots d\tau _k. \end{aligned} \end{aligned}$$

In the frequency domain, this can be presented as,

7 $$\begin{aligned} y(\omega ) = Y_1(\omega ) + Y_2(\omega ) + \cdots + Y_k(\omega ) \end{aligned}$$

where,

8 $$\begin{aligned} \begin{aligned} Y_k(\omega )&= \frac{1}{(2\pi )^{k-1}}\int _{0}^{\infty }\cdots \int _{0}^{\infty }H_k(\omega _1, \omega _2,\cdots \\&\cdots ,\omega -\omega _1-\omega _2-\cdots -\omega _k)X(\omega _1)X(\omega _2) \cdots \\&\cdots X(\omega - \omega _1 - \omega _2 - \cdots - \omega _k)d\omega _1d\omega _2\cdots d\omega _k \end{aligned} \end{aligned}$$

and $H_k$ is the HFRF of the $k^{th}$ level of nonlinearity.

If the Volterra series is extended to multiple degrees of freedom, the *k*-th order kernel must respect symmetry. For example, $h_k^{(p: a b \dots )}$ is invariant under permutations of its inputs and this simplifies the derivation. Without considering symmetry, the *k*-th order contribution at point *p* depends on all $n^k$ ordered input combinations, where *n* is the number of input locations. By accounting for symmetry, the number of unique input combinations reduces to $$\left( {\begin{array}{c}n+k-1\\ k\end{array}}\right) $$. Each of these represent a distinct way in which the kernel can mix *n* inputs at order *k*.

For the second-order contribution, subject to two separate simultaneous input contributions at points $x^{(a)}$ and $x^{(b)}$,

9 $$\begin{aligned} y^{(p)}_2(t)&= \int _{0}^{\infty }\int _{0}^{\infty }h_2^{(p:aa)}(\tau _1,\tau _2)x^{(a)}(t-\tau _1)x^{(a)}(t-\tau _2)d\tau _1d\tau _2 \nonumber \\&+ \int _{0}^{\infty }\int _{0}^{\infty }h_2^{(p:ab)}(\tau _1,\tau _2)x^{(a)}(t-\tau _1)x^{(b)}(t-\tau _2)d\tau _1d\tau _2 \nonumber \\&+ \int _{0}^{\infty }\int _{0}^{\infty }h_2^{(p:ba)}(\tau _1,\tau _2)x^{(b)}(t-\tau _1)x^{(a)}(t-\tau _2)d\tau _1d\tau _2 \nonumber \\&+ \int _{0}^{\infty }\int _{0}^{\infty }h_2^{(p:bb)}(\tau _1,\tau _2)x^{(b)}(t-\tau _1)x^{(b)}(t-\tau _2)d\tau _1d\tau _2, \end{aligned}$$

where it is evident that if the symmetric components are combined, repeating kernels can be reduced to,

10 $$\begin{aligned} h_2^{(p:ab)}(\tau _1,\tau _2) + h_2^{(p:ba)}(\tau _2,\tau _1)\rightarrow 2h_2^{(p:ab)}(\tau _1,\tau _2), \end{aligned}$$

which simplifies Eq. (9) to,

11 $$\begin{aligned} y^{(p)}_2(t)&= \int _{0}^{\infty }\int _{0}^{\infty }h_2^{(p:aa)}(\tau _1,\tau _2)x^{(a)}(t-\tau _1)x^{(a)}(t-\tau _2)d\tau _1d\tau _2 \nonumber \\&+ 2\int _{0}^{\infty }\int _{0}^{\infty }h_2^{(p:ab)}(\tau _1,\tau _2)x^{(a)}(t-\tau _1)x^{(b)}(t-\tau _2)d\tau _1d\tau _2 \nonumber \\&+ \int _{0}^{\infty }\int _{0}^{\infty }h_2^{(p:bb)}(\tau _1,\tau _2)x^{(b)}(t-\tau _1)x^{(b)}(t-\tau _2)d\tau _1d\tau _2. \end{aligned}$$

Like the $y_2$ contribution, the symmetry of higher-order kernels such as $h_3$ can be adopted to assist kernel derivation. In the case of $h_3$, when the input $x^{(a)}$ has two unique harmonics ($\tau _1$ and $\tau _2$), and $x^{(b)}$ is only made up of one ($\tau _3$), it follows that the kernels can be absorbed into,

12 $$\begin{aligned} \begin{aligned} 3h_3^{(p:aab)}(\tau _1,\tau _2,\tau _3)&= h_3^{(p:aab)}(\tau _1,\tau _2,\tau _3) \\&+ h_3^{(p:aba)}(\tau _1,\tau _3,\tau _2) + h_3^{(p:baa)}(\tau _3,\tau _2,\tau _1) \end{aligned}, \end{aligned}$$

whereas when a unique harmonic is applied at three different points, more variations exist,

13 $$\begin{aligned} \begin{aligned} 6h_3&^{(p:abc)}(\tau _1,\tau _2,\tau _3) = h_3^{(p:abc)}(\tau _1,\tau _2,\tau _3) \\&+ h_3^{(p:acb)}(\tau _1,\tau _3,\tau _2) + h_3^{(p:bac)}(\tau _2,\tau _1,\tau _3) \\  &+ h_3^{(p:bca)}(\tau _2,\tau _3,\tau _1) + h_3^{(p:cab)}(\tau _3,\tau _1,\tau _2) \\&+ h_3^{(p:cba)}(\tau _3,\tau _2,\tau _1). \end{aligned} \end{aligned}$$

### Harmonic probing

The harmonic simplifications assist in the application of the harmonic-probing algorithm of Bedrosian and Rice [28], extended to discrete systems by Billings and Tsang [29], then extended to MIMO Volterra series by Worden et al. [4]. As this paper explores all these algorithms to predict and validate the HFRFs of the chosen systems, a simple introduction is helpful in SISO to give context.

Considering a periodic excitation consisting of a single harmonic,

14 $$\begin{aligned} x(t) = e^{i\varOmega t}, \end{aligned}$$

a spectral representation can be created based on the Dirac $\delta$-function,

15 $$\begin{aligned} X(\omega ) = 2\pi \delta (\omega -\varOmega ), \end{aligned}$$

which helps to take advantage of the multiplication properties of the frequency-domain representation of the Volterra series over the more tedious convolution process in the time domain.

If Eq.  (15) is substituted into equations of the form (8) and integrated, using the frequency domain contribution Eq. (7), a relationship between the output FRF and the diagonals of the individual HFRFs of the system at each input harmonic can be obtained as,

16 $$\begin{aligned} \begin{aligned} Y(\omega )&= 2\pi \{H_1(\varOmega )\delta (\omega -\varOmega ) + H_2(\varOmega ,\varOmega )\delta (\omega -2\varOmega ) \\&+ H_3(\varOmega ,\varOmega ,\varOmega )\delta (\omega -3\varOmega )+\cdots \}, \end{aligned} \end{aligned}$$

where, taking the inverse Fourier transform gives a simplified relationship for the output time series,

17 $$\begin{aligned} \begin{aligned} y(t)&= H_1(\varOmega )e^{i\varOmega t} + H_2(\varOmega ,\varOmega )e^{i2\varOmega t} \\&+ H_3(\varOmega ,\varOmega ,\varOmega )e^{i3\varOmega t} + \cdots \end{aligned} \end{aligned}$$

This importantly shows that the output is made up of the multiplication of the input forcing and $H_1$ kernel transform, as well as the additional multiplications of higher-harmonic multiples of the input forcing. Equation (17) only presents information for the diagonals of each kernel. Depending on the truncation of the Volterra series to suitably predict the output, additional input “tones” are applied to the fully understand the properties of the HFRFs.

As another general expression for multiple periodic excitations (up to *k* separate tones), the input can be extended to give the form,

18 $$\begin{aligned} x(t) = e^{i\varOmega _1 t} + e^{i\varOmega _2 t} + \cdots + e^{i\varOmega _k t}, \end{aligned}$$

with frequency domain representation,

19 $$\begin{aligned} X(\omega ) \!=\! 2\pi \delta (\omega \!-\!\varOmega _1) \!+\! 2\pi \delta (\omega \!-\!\varOmega _2) \!+\! \cdots \!+\! 2\pi \delta (\omega \!-\!\varOmega _k).\nonumber \\ \end{aligned}$$

Functions of the form of Eq. (8) can then be manipulated to give relationships between the multiple input harmonics and the resultant output. A much more in-depth explanation of harmonic probing can be found in [5].

For a MIMO system excited by three different harmonics, using symmetry, one has three separate probing equations; each is simplified by rejecting contributions with higher multiples of harmonics such as $H_2(\varOmega _2, \varOmega _2)e^{i2\varOmega _2t}$.

20 $$\begin{aligned}  &   \begin{aligned} x^{(a)}(t) = e^{i\varOmega _1 t} + e^{i\varOmega _2 t} + e^{i\varOmega _3 t} \end{aligned} \end{aligned}$$

21 $$\begin{aligned}  &   \begin{aligned} y^{(p)}(t)&= H_1^{(p:a)}(\varOmega _1)e^{i\varOmega _1 t} + H_1^{(p:a)}(\varOmega _2)e^{i\varOmega _2 t} \\&+ H_1^{(p:a)}(\varOmega _3)e^{i\varOmega _3 t} \\&+ 2H_2^{(p:aa)}(\varOmega _1, \varOmega _2)e^{i(\varOmega _1 + \varOmega _2)t} \\&+ 2H_2^{(p:aa)}(\varOmega _1, \varOmega _3)e^{i(\varOmega _1 + \varOmega _3)t} \\&+ 2H_2^{(p:aa)}(\varOmega _2, \varOmega _3)e^{i(\varOmega _2 + \varOmega _3)t} \\&+ 6H_3^{(p:aaa)}(\varOmega _1, \varOmega _2, \varOmega _3)e^{i(\varOmega _1 + \varOmega _2 + \varOmega _3) t} +\cdots \end{aligned} \end{aligned}$$

22 $$\begin{aligned}  &   \begin{aligned} x^{(a)}(t)&= e^{i\varOmega _1 t} + e^{i\varOmega _2 t} \end{aligned} \end{aligned}$$

23 $$\begin{aligned}  &   \begin{aligned} x^{(b)}(t)&= e^{i\varOmega _3 t} \end{aligned} \end{aligned}$$

24 $$\begin{aligned}  &   \begin{aligned} y^{(p)}(t)&= H_1^{(p:a)}(\varOmega _1)e^{i\varOmega _1 t} + H_1^{(p:a)}(\varOmega _2)e^{i\varOmega _2 t} \\&+ H_1^{(p:b)}(\varOmega _3)e^{i\varOmega _3 t} \\&+ 2H_2^{(p:aa)}(\varOmega _1, \varOmega _2)e^{i(\varOmega _1 + \varOmega _2)t} \\&+ 2H_2^{(p:ab)}(\varOmega _1, \varOmega _3)e^{i(\varOmega _1 + \varOmega _3)t} \\&+ 2H_2^{(p:ab)}(\varOmega _2, \varOmega _3)e^{i(\varOmega _2 + \varOmega _3)t} \\&+ 6H_3^{(p:aab)}(\varOmega _1, \varOmega _2, \varOmega _3)e^{i(\varOmega _1 + \varOmega _2 + \varOmega _3) t} +\cdots \end{aligned} \end{aligned}$$

25 $$\begin{aligned}  &   \begin{aligned} x^{(a)}(t)&= e^{i\varOmega _1 t} \end{aligned} \end{aligned}$$

26 $$\begin{aligned}  &   \begin{aligned} x^{(b)}(t)&= e^{i\varOmega _2 t} \end{aligned} \end{aligned}$$

27 $$\begin{aligned}  &   \begin{aligned} x^{(c)}(t)&= e^{i\varOmega _3 t} \end{aligned} \end{aligned}$$

28 $$\begin{aligned}  &   \begin{aligned} y^{(p)}(t)&= H_1^{(p:a)}(\varOmega _1)e^{i\varOmega _1 t} + H_1^{(p:b)}(\varOmega _2)e^{i\varOmega _2 t} \\&+ H_1^{(p:c)}(\varOmega _3)e^{i\varOmega _3 t} \\&+ 2H_2^{(p:ab)}(\varOmega _1, \varOmega _2)e^{i(\varOmega _1 + \varOmega _2)t} \\&+ 2H_2^{(p:ac)}(\varOmega _1, \varOmega _3)e^{i(\varOmega _1 + \varOmega _3)t} \\&+ 2H_2^{(p:bc)}(\varOmega _2, \varOmega _3)e^{i(\varOmega _2 + \varOmega _3)t} \\&+ 6H_3^{(p:abc)}(\varOmega _1, \varOmega _2, \varOmega _3)e^{i(\varOmega _1 + \varOmega _2 + \varOmega _3) t} +\cdots \end{aligned} \end{aligned}$$

Using the three derived Eqs. (21), (24) and (28), the equation of motion or recovered weighted neural network equation for a specific nonlinear system can be probed to separate the linear and nonlinear kernels in the frequency domain for inspection-up to the third order in this case [5].

### MIMO NARX neural-network structure

When forecasting a nonlinear-system time series, very often the Nonlinear Auto-Regressive Moving Average with eXogenous inputs (NARMAX) [30] approach is adopted because of its versatility and simplicity. This approach uses the discrete ‘lagged’ inputs, outputs and the noise of the model over a specified duration to help inform the output at the following time step. If one assumes that the noise involved is white Gaussian, the ‘Moving Average’ part can be ignored, and one is left with the simpler NARX model highlighted in Eq. (29), where in this case, $y_t^*$ is the current time step prediction, and the inputs ‘*x*’ and outputs ‘*y*’ are lagged up to $n_x$ and $n_y$ respectively,

29 $$\begin{aligned} y_t^* = F(y_{t-1},\cdots ,y_{t-n_y}, x_{t},\cdots ,x_{t-(n_x -1)}) + \epsilon _t, \end{aligned}$$

where the equation considers a nonlinear function model with *n* degrees of freedom that relates all the inputs and exogenous inputs to all the output channels available.

Equation (30) presents a specific function that relates all the lagged inputs and exogenous inputs to channel *p*,

30 $$\begin{aligned} \begin{aligned} y_t^{(p)*}&= F^{(p)}(y^{(1)}_{t-1},\cdots ,y^{(1)}_{t-n_y}, x^{(1)}_{t},\cdots ,x^{(1)}_{t-(n_x -1)},\cdots \\&\cdots ,y^{(n)}_{t-1},\cdots ,y^{(n)}_{t-n_y}, x^{(n)}_{t},\cdots ,x^{(n)}_{t-(n_x -1)}). \end{aligned} \end{aligned}$$

The multi-layer perceptron (MLP) neural network is adopted here to train the data to forecast the multiple output-channel data, each having a different functional relationship to the many input channels [31, 32]. This relationship is dictated only by the output weights connecting individual output channels to the hidden-layer neurons. Therefore, if it was possible to probe the NARX neural network representing a SDOF system, the NARX NN could also be probed to recover the HFRFs for all the input and output relationships up to the order the model permits by splitting up the input and output locations.

31 $$\begin{aligned} y^{(p)}_t&= \sum _{i=0}^{\infty } \sum _{j=0}^{n_h} \frac{w_j^{(p)}{\tanh ^{(i)}(b_j)}}{i!}\Big (\sum _{m = 0}^{n_x-1}u^{(1)}_{jm}x^{(1)}_{t-m} + \cdots \nonumber \\&+ \sum _{m=0}^{n_x-1}u^{(n)}_{jm}x^{(n)}_{t-m} + \sum _{k = 1}^{n_y}v^{(1)}_{jk}y^{(1)}_{t-k} + \cdots \nonumber \\&+ \sum _{k=1}^{n_y}v^{(n)}_{jk}y^{(n)}_{t-k} \Big )^i \end{aligned}$$

The Eq. (31) presents the relationship between input and output at point *p* that can be probed to recover the different frequency-domain kernels. The equation, as in the work by Wray and Green [2] and Chance et al. [3], uses the Taylor expansion of the activation function to separate the output contributions by order; this will recover the linear, quadratic, cubic and other higher-order contributions for a variety of input–output relationships.

**Fig. 1:**
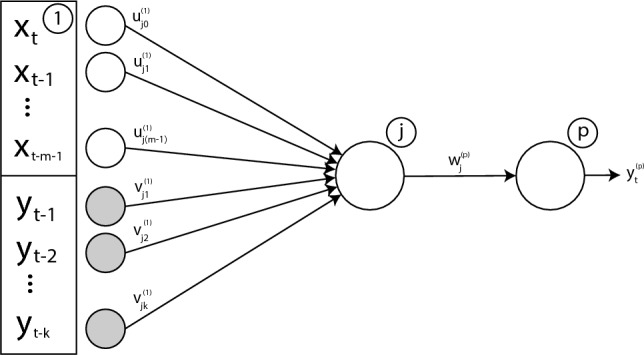
Example of lag and weight channel structure

As can be seen in Fig. 1, the channels for individual exogenous inputs and inputs (white and grey circles) consist of discrete ‘lagged’ data, multiplied by the specific weights and combined at hidden neuron ‘j’. Since the exogenous inputs are defined one time step ahead of the inputs to predict the current time-step output, it is necessary to assign different letters ‘*m*’ and ‘*k*’ to the input and exogenous lag parameters respectively. It proved beneficial here to keep the number of lags equal; this greatly simplifies the NARX NN equations and had comparable accuracy to alternating the hyperparameter.

**Fig. 2:**
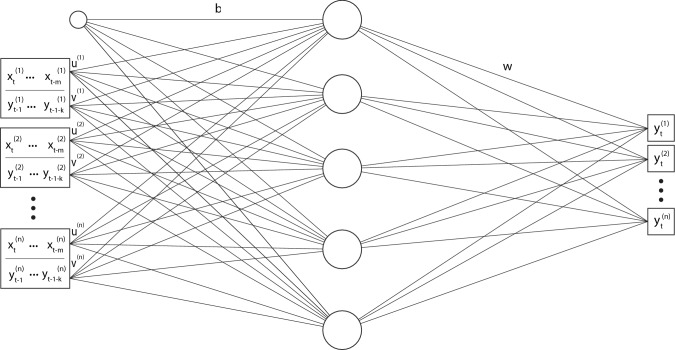
Example MIMO NARX neural-network structure

The complexities of the individual mode input blocks are presented in Fig. 1, which depicts the flow of the input block ‘1’ through individual hidden neuron ‘j’, contributing to the output from channel ‘p’. For simplicity, Fig. 2 only presents one lag for each input component of each node.

This full NARX neural-network structure is presented in Fig. 2, to show the weights relating the bias ‘*b*’, exogenous inputs ‘*u*’ and inputs ‘*v*’ to the hidden nodes, and the weights ‘*w*’ relating the hidden nodes to the outputs. Each specific input also represents a pre-defined time lag from all the input channels, combined at the hidden nodes, run through an activation function (Taylor expansion of Tanh) and combined at each output channel to forecast the system like the Volterra series, here with multiple input and output-channels.

Using Eq. (30), for each time-step combination of lagged inputs and substituting into the function *F*, the corresponding output at that timestep can be predicted, where *F* relates to the neural network. This provides a One-Step-Ahead (OSA) prediction to determine prediction accuracy. This approach is flawed as it is difficult to determine if a model has been overfit. A more thorough implementation is the Model-Predicted-Output (MPO), presented in Eq. (32), where the predicted outputs denoted with an asterisk (*) are substituted back into the function as inputs, therefore propagating prediction error forward and leading to a more meaningful prediction.

32 $$\begin{aligned} \begin{aligned} y_t^* = F(y^{(1)*}_{t-1},&\cdots ,y^{(1)*}_{t-n_y}, x^{(1)}_{t},\cdots ,x^{(1)}_{t-(n_x -1)},\cdots \\&\cdots ,y^{(n)*}_{t-1},\cdots ,y^{(n)*}_{t-n_y}, x^{(n)}_{t},\cdots ,x^{(n)}_{t-(n_x -1)}) \end{aligned} \end{aligned}$$

The MPO approach was therefore adopted for this research and the *Normalised Mean Squared Error* NMSE metric was used to quantify the error. The NMSE is defined as,

33 $$\begin{aligned} NMSE(y^*) = \frac{100}{N\sigma _y^2}\sum _{i=1}^{N}(y_i-y_i^*)^2. \end{aligned}$$

The Eq. (33) normalises each data channel with respect to the variance $\sigma _y$ to generate a more consistent comparison of measurements between adjacent channels. An average of these channel NMSE predictions therefore quantifies the total prediction accuracy of the network. Typically, an NMSE below 5.0 can be considered a good fit, whereas anything below 1.0 can be considered to be an excellent agreement.

## Applying MIMO volterra series theory

The systems chosen to demonstrate recovery of nonlinear contributions were simple spring-mass-damper systems with additional quadratic and cubic springs like the asymmetric Duffing oscillator, only connected between the masses 1 and *n* to the walls and not to adjacent masses, simplifying the theoretical equations to compare. Equations were derived to account for systems up to ‘*n*’ arbitrary orders. This helps to present how the accuracy of neural network prediction is affected by increasing order. The data generated for this paper were purely synthetic. Orders up to 5DOF were considered with varying noise levels for analysis.

**Fig. 3:**
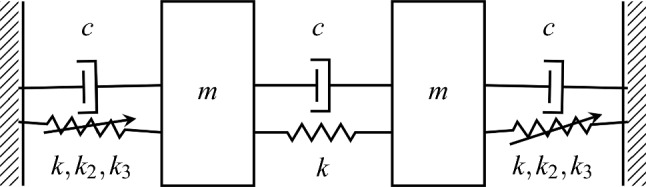
2DOF nonlinear system

For the 2DOF system presented in Fig. 3, the following equations of motions can be derived,

34 $$\begin{aligned}  &   m\ddot{y}^{(1)} + 2c\dot{y}^{(1)} - c\dot{y}^{(2)} + 2ky^{(1)} - ky^{(2)} \nonumber \\  &   \quad + k_2{y^{(1)}}^2 + k_3{y^{(1)}}^3 = x^{(1)}(t) \end{aligned}$$

35 $$\begin{aligned}  &   m\ddot{y}^{(2)} + 2c\dot{y}^{(2)} - c\dot{y}^{(1)} + 2ky^{(2)} - ky^{(1)} \nonumber \\  &   \quad + k_2{y^{(2)}}^2 + k_3{y^{(2)}}^3 = x^{(2)}(t). \end{aligned}$$

As seen in [4], for the first-order kernel, the probing inputs required are represented by a single harmonic at *p* and a lack of input at *q*, so $n=1$ and $k=1$ gives one unique kernel input combination from $\frac{1!}{1!0!}$,

36 $$\begin{aligned} x^{(p)}(t)= &   e^{i\varOmega t}, \end{aligned}$$

37 $$\begin{aligned} x^{(q)}= &   0. \end{aligned}$$

giving output contributions only accounting for input *p*,

38 $$\begin{aligned} y^{(p)}(t)= &   H_1^{(p:p)}(\varOmega )e^{i\varOmega t}, \end{aligned}$$

39 $$\begin{aligned} y^{(q)}(t)= &   H_1^{(q:p)}(\varOmega )e^{i\varOmega t}. \end{aligned}$$

Therefore, by replacing the parameters in Eqs. (34) and (35) by these inputs and outputs, one can derive expressions for the linear kernels $H_1$ in the frequency domain [4]:

40 $$\begin{aligned} \begin{aligned}&\mathbf {H_1}^{(2)}(\varOmega ) = \begin{bmatrix} H_1^{(1:1)}(\varOmega ) &  H_1^{(1:2)}(\varOmega ) \\ H_1^{(2:1)}(\varOmega ) &  H_1^{(2:2)}(\varOmega ) \\ \end{bmatrix} \\&=\begin{bmatrix} \begin{array}{c}-m\varOmega ^2 + 2k +2ci\varOmega \end{array} &  -ci\varOmega - k \\ -ci\varOmega - k &  \begin{array}{c}-m\varOmega ^2 + 2k + 2ci\varOmega \end{array} \\ \end{bmatrix}^{-1}. \end{aligned} \end{aligned}$$

**Fig. 4:**
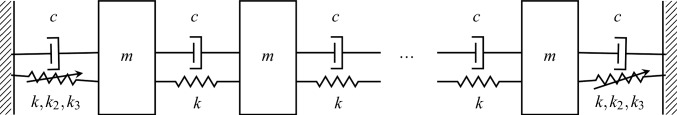
*n* DOF nonlinear system

For a system of *n* degrees of freedom, as in Fig. 4, the matrix of $H_1$’s can be derived using matrix inversion as,

41 $$\begin{aligned} \begin{aligned} \mathbf {H_1^{(n)}}(\varOmega )&= \begin{bmatrix} H_1^{(1:1)}(\varOmega ) &  H_1^{(1:2)}(\varOmega ) &  \cdots &  H_1^{(1:n)}(\varOmega ) \\ H_1^{(2:1)}(\varOmega ) &  H_1^{(2:2)}(\varOmega ) &  \cdots &  H_1^{(2:n)}(\varOmega ) \\ \vdots &  \vdots &  \ddots & \vdots \\ H_1^{(n:1)}(\varOmega ) &  H_1^{(n:2)}(\varOmega ) &  \cdots &  H_1^{(n:n)}(\varOmega ) \end{bmatrix} \\&=\begin{bmatrix} \begin{array}{c}-m\varOmega ^2 + 2k\\ +2ci\varOmega \end{array} &  -ci\varOmega - k &  \cdots &  0 \\ -ci\varOmega - k &  \begin{array}{c}-m\varOmega ^2 + 2k\\ +2ci\varOmega \end{array} &  \cdots &  0 \\ \vdots &  \vdots &  \ddots & \vdots \\ 0 &  0 &  \cdots &  \begin{array}{c}-m\varOmega ^2 + 2k\\ +2ci\varOmega \end{array} \end{bmatrix}^{-1} \end{aligned}. \end{aligned}$$

This approach can be repeated for $H_2$; however, since there are more input combinations, these pairs need to be accounted for to fully understand the contribution to the output.

These additional input combinations include,

42 $$\begin{aligned} x^{(p)}(t)= &   e^{i\varOmega _1 t} + e^{i\varOmega _2 t}, \end{aligned}$$

43 $$\begin{aligned} x^{(q)}(t)= &   0, \end{aligned}$$

which give outputs of,

44 $$\begin{aligned} \begin{aligned} y^{(p)}(t)&= H_1^{(p:p)}(\varOmega _1)e^{i\varOmega _1 t} + H_1^{(p:p)}(\varOmega _1)e^{i\varOmega _2 t} \\&+ 2H_2^{(p:pp)}(\varOmega _1,\varOmega _2)e^{i(\varOmega _1 +\varOmega _2) t}, \end{aligned} \end{aligned}$$

45 $$\begin{aligned} \begin{aligned} y^{(q)}(t)&= H_1^{(q:p)}(\varOmega _1)e^{i\varOmega _1 t} + H_1^{(q:p)}(\varOmega _1)e^{i\varOmega _2 t}\\&+ 2H_2^{(q:pp)}(\varOmega _1,\varOmega _2)e^{i(\varOmega _1 +\varOmega _2) t}. \end{aligned} \end{aligned}$$

Also, the cross combination of inputs can be used, which consists of inputs,

46 $$\begin{aligned} x^{(p)}(t)= &   e^{i\varOmega _1 t}, \end{aligned}$$

47 $$\begin{aligned} x^{(q)}(t)= &   e^{i\varOmega _2 t}, \end{aligned}$$

leading to output combinations of,

48 $$\begin{aligned}  &   \begin{aligned} y^{(p)}(t)&= H_1^{(p:p)}(\varOmega _1)e^{i\varOmega _1 t} + H_1^{(p:q)}(\varOmega _1)e^{i\varOmega _2 t} \\&+ 2H_2^{(p:pq)}(\varOmega _1,\varOmega _2)e^{i(\varOmega _1 +\varOmega _2) t}, \end{aligned} \end{aligned}$$

49 $$\begin{aligned}  &   \begin{aligned} y^{(q)}(t)&= H_1^{(q:p)}(\varOmega _1)e^{i\varOmega _1 t} + H_1^{(q:q)}(\varOmega _1)e^{i\varOmega _2 t} \\&+ 2H_2^{(q:pq)}(\varOmega _1,\varOmega _2)e^{i(\varOmega _1 +\varOmega _2) t}. \end{aligned} \end{aligned}$$

These newly-found equations can be used to probe the same equations of motion to recover additional $H_2$ kernels by noticing the coefficient of $H_2$ matrix in the derivation are equivalent to manipulations of the inverse of the already derived $H_1$ kernel matrix,

50 $$\begin{aligned} \begin{aligned}&\begin{bmatrix} H_2^{(1:11)}(\varOmega _1,\varOmega _2) &  \cdots &  H_2^{(1:nn)}(\varOmega _1,\varOmega _2) \\ \vdots &  \ddots & \vdots \\ H_2^{(n:11)}(\varOmega _1,\varOmega _2) &  \cdots &  H_2^{(n:nn)}(\varOmega _1,\varOmega _2) \end{bmatrix} \\&=\mathbf {H_1^{(n)}}(\varOmega _1 + \varOmega _2) \begin{bmatrix} \alpha ^{(1:11)} &  \alpha ^{(1:12)} &  \cdots &  \alpha ^{(1:nn)} \\ \alpha ^{(2:11)} &  \alpha ^{(2:12)} &  \cdots &  \alpha ^{(1:nn)} \\ \vdots &  \vdots &  \ddots &  \vdots \\ \alpha ^{(n:11)} &  \alpha ^{(n:12)} &  \cdots &  \alpha ^{(n:nn)} \\ \end{bmatrix}, \end{aligned} \end{aligned}$$

with

51 $$\begin{aligned} \alpha ^{(j:pq)}&= -(b)k_2 H_1^{(j:p)}(\varOmega _1)H_1^{(j:q)}(\varOmega _2), \end{aligned}$$

where $b = 1$ if $j = 1$ or n; otherwise $b = 0$, since there exist no nonlinear spring connections between masses.

The same process can be repeated for higher-order kernels, such as the $H_3$ kernel. These equations relate specifically to the *n*-DOF system presented in Fig. 4,

52 $$\begin{aligned} \begin{aligned}&\begin{bmatrix} H_3^{(1:111)}(\varOmega _1,\varOmega _2,\varOmega _3) &  \cdots &  H_3^{(1:nnn)}(\varOmega _1,\varOmega _2,\varOmega _3) \\ \vdots &  \ddots & \vdots \\ H_3^{(n:111)}(\varOmega _1,\varOmega _2,\varOmega _3) &  \cdots &  H_3^{(n:nnn)}(\varOmega _1,\varOmega _2,\varOmega _3) \end{bmatrix} \\&=\mathbf {H_1^{(n)}}(\varOmega _1 + \varOmega _2 + \varOmega _3) \begin{bmatrix} \alpha ^{(1:111)} &  \alpha ^{(1:112)} &  \cdots &  \alpha ^{(1:nnn)} \\ \alpha ^{(2:111)} &  \alpha ^{(2:112)} &  \cdots &  \alpha ^{(1:nnn)} \\ \vdots &  \vdots &  \ddots &  \vdots \\ \alpha ^{(n:111)} &  \alpha ^{(n:112)} &  \cdots &  \alpha ^{(n:nnn)} \\ \end{bmatrix} \end{aligned} \end{aligned}$$

and

53 $$\begin{aligned} \alpha ^{(j:pqr)}&= -b(\frac{2}{3}k_2(H_1^{(j:p)}(\varOmega _1)H_2^{(j:qr)}(\varOmega _2,\varOmega _3)\nonumber \\&+ H_1^{(j:q)}(\varOmega _2)H_2^{(j:pr)}(\varOmega _1,\varOmega _3)\nonumber \\&+ H_1^{(j:r)}(\varOmega _3)H_2^{(j:pq)}(\varOmega _1,\varOmega _2))\nonumber \\&-k_3(H_1^{(j:p)}(\varOmega _1)H_1^{(j:q)}(\varOmega _2)H_1^{(j:r)}(\varOmega _3))), \end{aligned}$$

with this derivation explained more thoroughly in Appendix B. As in the previous example in Eq. (51) $b = 1$ if $j = 1$ or *n*, otherwise b = 0. If the positions of nonlinearities are altered, a new equation of motion must be probed, lending to the requirement of a simpler more versatile HFRF recovery method.

Since it has been shown that the equation of motion can be probed to recover the different kernel orders, the procedure can be modified to work for discrete-time models such as the NARX neural network model presented earlier. Since this model is discrete, a manipulation of the lagged input and output components must be assembled to adapt equations like (21), (24) and (28) to probe the neural network Eq. (31) and extract the harmonic components. The complexity of these derivation supports the adoption of the NARX NN architecture to recover the kernels of the system [3]. For each defined time lag *m*, the input can be reduced to,

54 $$\begin{aligned} x_{t-m} = e^{i\varOmega (t-m\delta t)}, \end{aligned}$$

which shows that for an input at one point, using output time lag *k*, the output is made of,

55 $$\begin{aligned} y_{t-k} = H_1(\varOmega )e^{i\varOmega (t-k\delta t)}. \end{aligned}$$

Because of the length of the algebra, the recovery method of the kernels $H_1$ to $H_3$ has also been presented in Appendix C. The matrices generated have great importance, as they show that neural network systems with input–output relationships up to *n* orders can be probed to recover all the frequency-domain output contributions the architecture provides. This is impressive since, for time-invariant systems of unknown nonlinearity, the NN can be trained to sufficiently forecast the responses as well as recover the linear and nonlinear HFRF kernels relating input–output data channels. The recovery of the HFRFs eliminates the need to know any equations of motion that make up the system and therefore, the confidence of both the forecasted output data and generated kernels transforms can provide insight into these complicated systems as well as confidence of their legitimacy.

## Results

Since the theory showed it was possible to recover MIMO Volterra kernels using the NARX NN structure, it was implemented for a 2DOF and 5DOF system like Fig. 4. The linear parameters were chosen as $m = 1kg$, $c = 5Ns^{-1}$ and $k = 10^4Nm^{-1}$. The nonlinear parameters used were $k_2 = 10^7Nm^{-2}$, $k_3 = 5 \times 10^9Nm^{-3}$. These parameters were commonly adopted in literature [5] so the Volterra orders each had sufficient output contribution, not dominated by a specific order. For the systems in Fig. 4, symmetries are present, making specific kernels equivalent, like the 2DOF example, $H_1^{(1:1)} = H_1^{(2:2)}$. For ease of visualisation, repeating kernels are not included. Training the NARX NN, output data were generated over 20 s with time step 0.0005 s in state-space using fourth-order Runge–Kutta and first-order-hold. These data were subsampled to a time step of 0.0025 s (sampling frequency of 400Hz), to sufficiently train the network for different noise intensities and DOFs. The system was forced using a random-phase multisine input with RMS (root-mean-square) of 2N with a bandwidth of (0–50Hz) to simulate Gaussian white noise over that interval. The Taylor expansion of the activation function was limited to third order, also commonly adopted in literature [5] matching the highest known nonlinearity in the system. Truncating at a higher-order would present convergence issues from the Taylor expansion and important system information like harmonics are found up to order three. Hyperparameters were chosen by altering both input and output kernel sized separately, leading to the decision of equal kernel sizes. The results in Appendix A show minimal benefits, which simplified the recovery equations greatly. Weights were randomised, where optimal trained weights giving smallest validation error were restored to probe the network with.

### 2DOF system

The kernels for the nonlinear 2DOF system represented by Fig. 3 were extracted using the equations in Appendix C. The hyperparameters chosen were kernel size = 10 for both input and output, learning rate = $1 \times 10^{-2}$ and one hidden layer with size = 32. The network was trained using Adam gradient descent, implemented in PyTorch with no weight decay, batch size = 32 and activation function ‘Tanh’. The hyperparameter justification is presented in Appendix A.

**Fig. 5:**
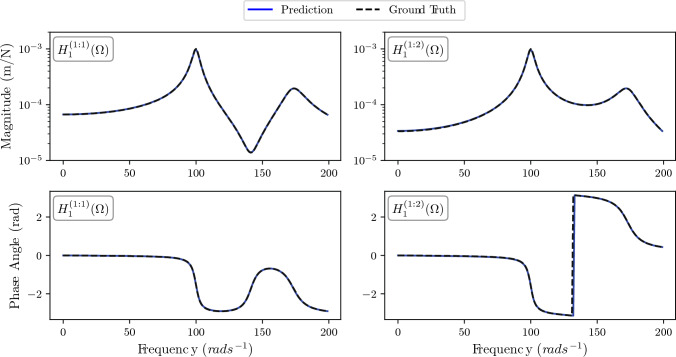
Comparison of $H_1$ kernel predictions for 2DOF system (0% noise)

**Fig. 6:**
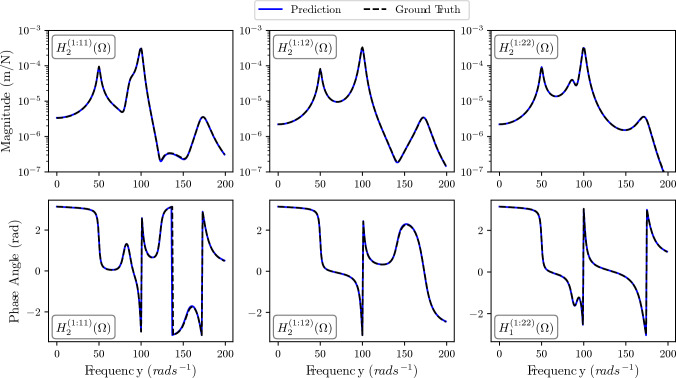
Comparison of $H_2$ kernel predictions diagonal for 2DOF system (0% noise)

**Fig. 7:**
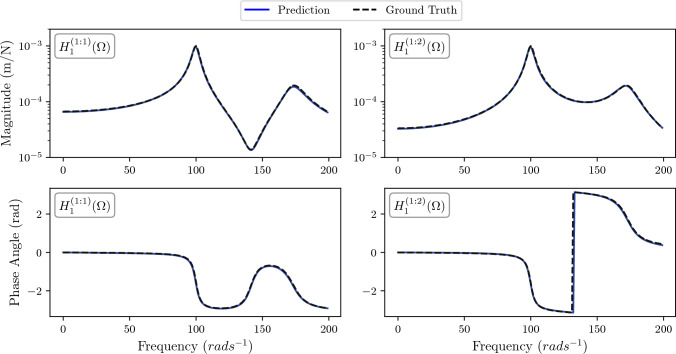
Comparison of $H_1$ kernel predictions for 2DOF system (5% noise)

**Fig. 8:**
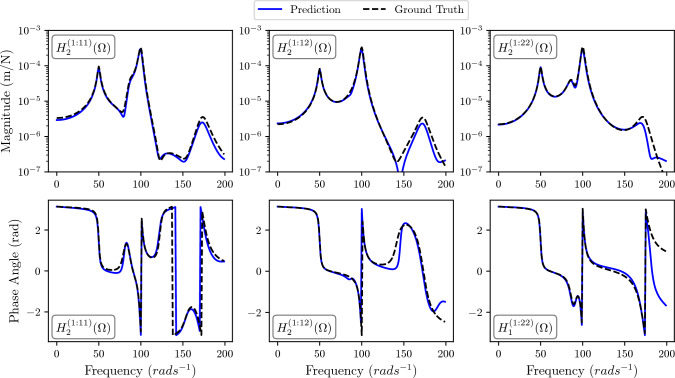
Comparison of $H_2$ kernel diagonal predictions for 2DOF system (5% noise)

Each figure is labelled as $H_1^{(p:q)}(\varOmega )$, where *p* and *q* represent input and output location respectively. As can be seen in Fig. 5 the agreement between prediction and Ground Truth is strong, proving the neural network was capable of separating response contributions. This prediction was complimented by low MPO NMSE and MAE for the test dataset predictions (0.01867 and 0.008691 to four significant figures respectively). Predictions are well below 1.0, indicating excellent agreements, proving the network was sufficiently trained. For the $H_2$ kernel recovery in Fig. 6, although the small error in the $H_1$ kernel was carried forward, the weights of the neural network were still capable of reconstructing the HFRFs based on the training data. Therefore, 5% RMS noise was added to the input and output training and validation data to test how robust the neural network was at constructing the HFRFs (Figs. 7 and 8).

The additional noise led to an expected decrease in accuracy; however, the structure of the kernels remained clear. The MPO NMSE and MAE for the test dataset predictions were 1.30093 and 0.07003, which indicated a drop, but still good agreement. For receptance FRFs, the amplitude of the modes falls with frequency, so ‘signal-to-noise’ decreases with frequency; therefore, one might expect the accuracy of the recovery to fall with increasing frequency.

**Fig. 9:**
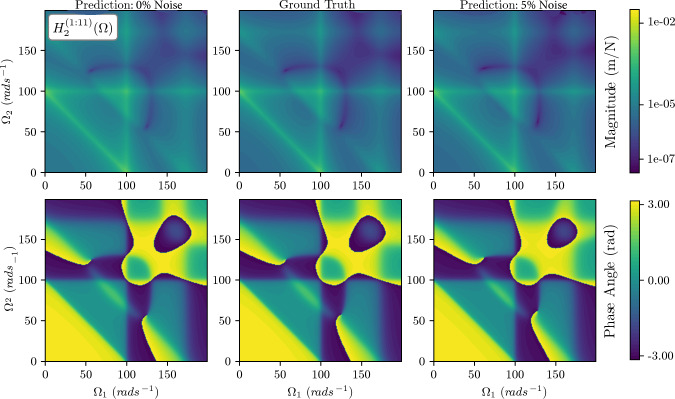
Comparison of $H_2^{(1:11)}$ kernel predictions, 0% noise (left), ground truth (middle), 5% noise (right)

**Fig. 10:**
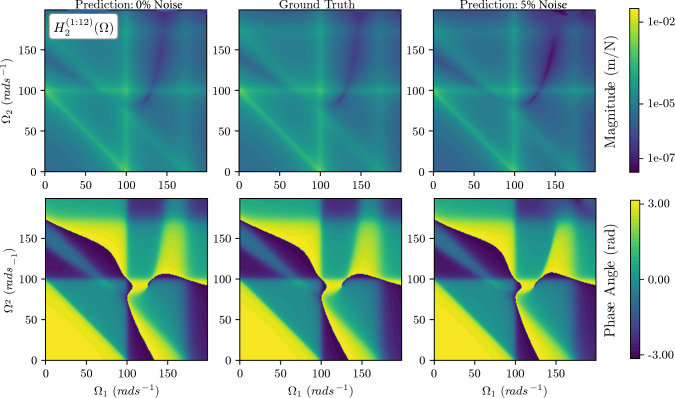
Comparison of $H_2^{(1:12)}$ kernel predictions, 0% noise (left), ground truth (middle), 5% noise (right)

**Fig. 11:**
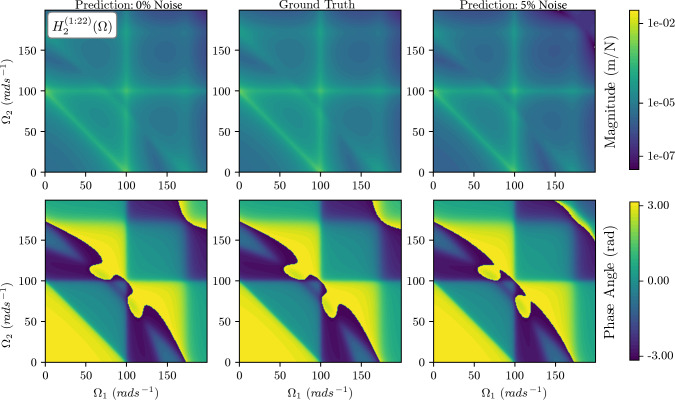
Comparison of $H_2^{(1:22)}$ kernel predictions, 0% noise (left), ground truth (middle), 5% noise (right)

The $H_2$ kernels are plotted in 2D contour form in Figs. 9, 10 and 11, displaying the difference between noise levels compared with theoretical predictions. The difference in results when noise was added is more noticeable in the phase plots on the lower row of Fig. 11, whereas the differences in predicted magnitude were hard to distinguish. The overall shape is clearly correct, suggesting a good estimate like in the HFRF’s diagonal predictions.

**Fig. 12:**
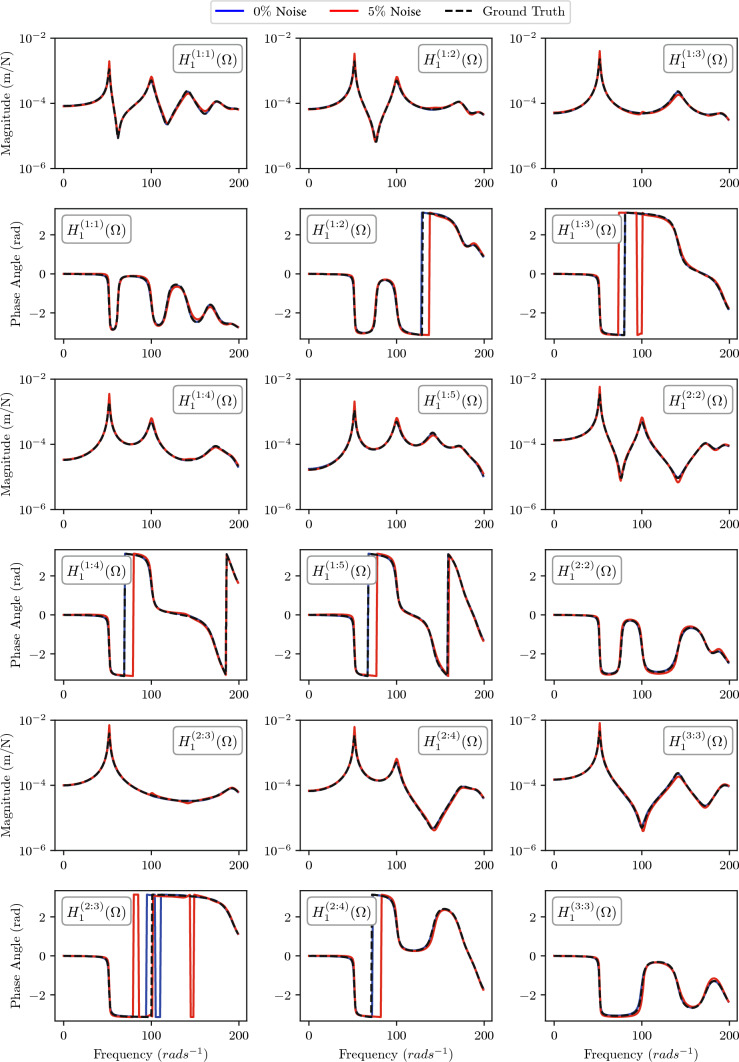
Comparison of all the unique $H_1$ kernel predictions for 5DOF system

### 5DOF system

Since it was evident that the NARX NN structure can recover the Volterra kernels of an *n*-DOF system, the number of degrees of freedom present was increased to 5DOF. This was done not only to showcase the generality of neural networks, but also to prove that the derived ‘*n*-degree-of-freedom’ formulae could robustly recover the increased number of HFRFs. The data-generation parameters, such as frequency and timespan, for training the 5DOF system were kept the same as for the 2DOF system, only with the obvious alteration of the equations of motion simulation equations to allow five channels, promoting consistent predictions. The kernel size remained at ten as well as the hidden-unit size of 32. The hyperparameter search yielded similar results to the 2DOF case by comparing combinations of learning rates, lags, and hidden neurons. Although the parameters were kept the same, this choice of structure together with the fact there were five sets of input and output channels meant that it was difficult to sufficiently train the network until enough data were collected to avoid the system from being overfitted. Therefore, it is important that the network is trained sufficiently as otherwise the predicted kernels would bear poor resemblance to the truth, even if the training data suggests the predictions made were excellent.

As the order of the system increases, more input–output relationships exist. In the case of a 5DOF system, 25 kernels exist leading to 50 plots for phase and magnitude. Since the chosen system was symmetric, one can use this assumption alongside the reciprocity rule for the linear portion of the system to reduce the output to nine unique HFRFs. The 0% and 5% noise predictions were plotted together with ground truth for comparison.

As can be seen in Fig. 12, the quality of results seen for the 2DOF case was also seen in these predictions, thereby highlighting how robust the NARX NN architecture is in forecasting the system, as the kernels that describe it were consistently encoded within the networks weights.

**Fig. 13:**
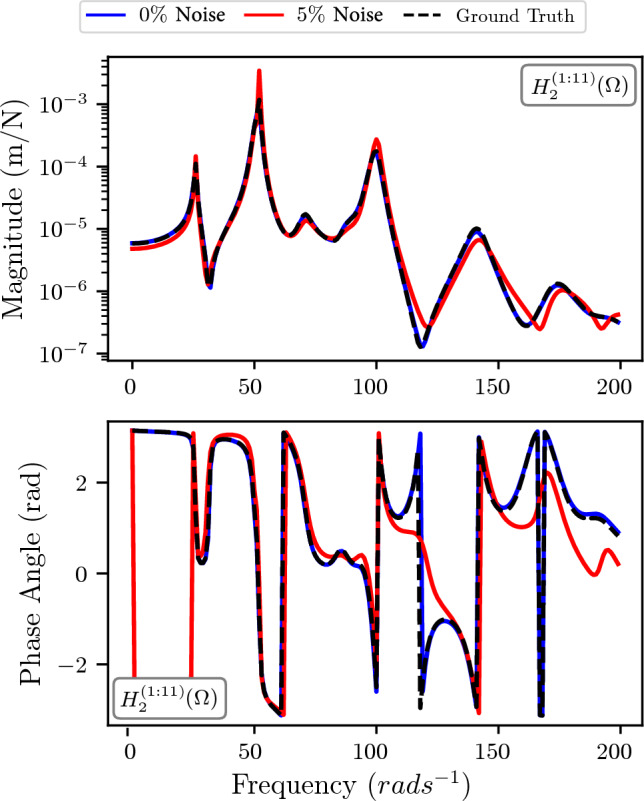
Comparison of $H_2^{(1:11)}$ diagonal kernel predictions for 5DOF system

**Fig. 14:**
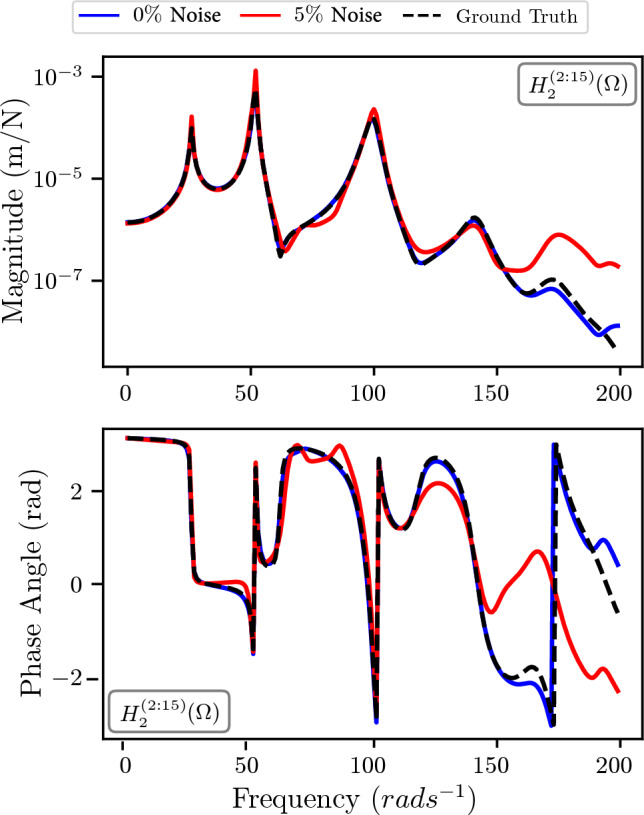
Comparison of $H_2^{(2:15)}$ diagonal kernel predictions for 5DOF system

**Fig. 15:**
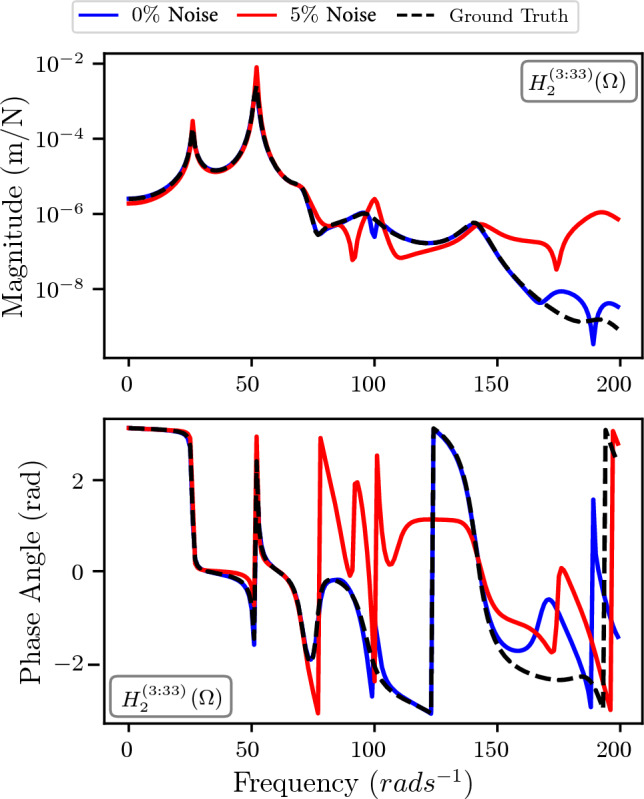
Comparison of $H_2^{(3:33)}$ diagonal kernel predictions for 5DOF system

Like plotting the FRFs for the 5DOF $H_1$ kernels, replicating this for $H_2$ would produce 150 plots, and even a reduction via symmetry would not lead to a much better visualisation. Therefore, three input–output combinations (after observing the results) were chosen to indicate the impact of recovering kernels throughout the system (Figs. 13, 14, 15). These specific chosen plots had noticeably varying prediction accuracy, allowing for further discussion of the reasons for this and possible limitations for the algorithm as to whether these differences highlight if the output-channel predictions could be improved.

Interestingly, measuring the responses closer to the location of the nonlinearity shown in Fig. 13 led to better predictions for the $H_2$ kernels than measuring further away, such as the excitation and displacement of the centre mass in Fig. 15. The predictions for all were consistent for frequency predictions up to 150 $rads^{-1}$ even for complicated kernel shapes, such as $H_2^{(1:11)}(\varOmega )$. This result, therefore, suggested that the model struggles to predict kernels where the influence of the nonlinearity is weak. As expected, the 0% noise predictions were much more consistent, where the predicted structure of kernels which are influenced less by nonlinearities, still had a good structure compared to that of the 5% noise predictions which deviated from ground truth at higher frequencies. One can therefore assume that running multiple training iterations with different initial weights could eventually give an average kernel prediction that matched that of the ground truth. The MPO NMSE and MAE for test datasets with 0% noise were 0.09914 and 0.01996, whereas with 5% noise the errors were 4.87546 and 0.16538, which were still good predictions. However, this also indicated how difficult it is to train a network and predict the output for such a system. For more complex systems, the shapes of the HFRF could really help in constraining models whilst training to help predict when data is limited.

**Fig. 16:**
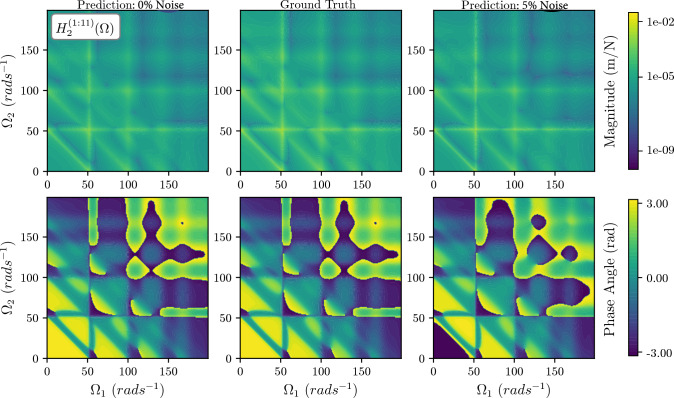
Comparison of $H_2^{(1:11)}$ kernel predictions for 5DOF system, 0% noise (left), ground truth (middle), 5% noise (right)

**Fig. 17:**
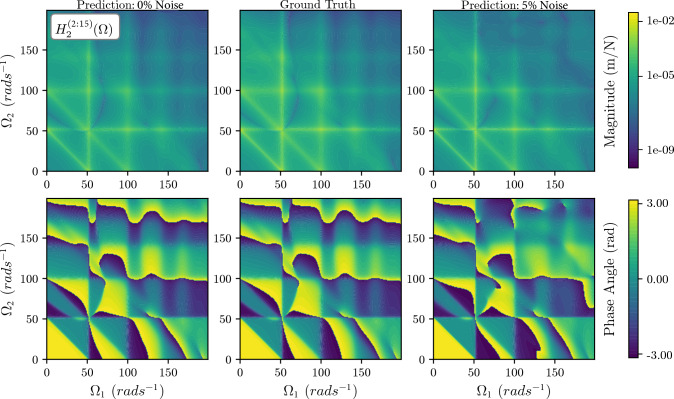
Comparison of $H_2^{(2:15)}$ kernel predictions for 5DOF system, 0% noise (left), ground truth (middle), 5% noise (right)

**Fig. 18:**
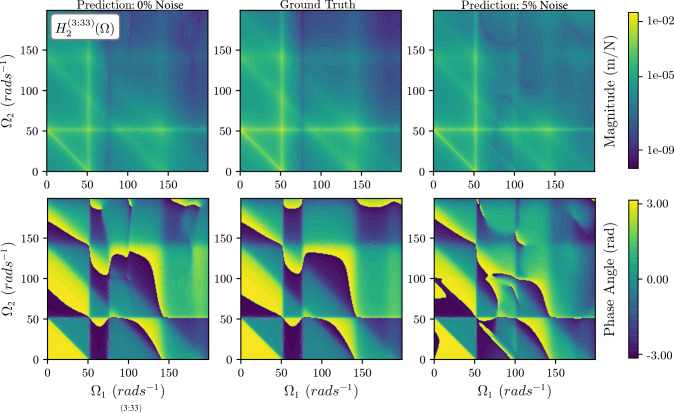
Comparison of $H_2^{(3:33)}$ kernel predictions for 5DOF system, 0% noise (left), ground truth (middle), 5% noise (right)

As mentioned previously, many other plots were constructed for each input–output combination, which gave prediction accuracies consistent with those seen in Figs. 13, 14 and 15. Therefore, the 2D plots were also constructed for these predictions of the 5DOF kernel transforms to observe how the constructed magnitude and phase looked for different frequency combinations (Figs. 16, 17, 18). The phase plots for the $H_2$ kernel predictions were again a much better indication of the prediction consistency compared with the magnitude. This can be seen in all three figures, where the construction of the 0% noise kernels was very consistent. Comparing this with the 5% noise prediction, the phase structure was still there, but with much less consistency, especially at higher frequencies. Importantly, the kernel predictions when the magnitudes were largest are the most accurate, where both sets of kernel predictions show good agreement with the theoretical prediction. Since a sampling frequency of 400Hz was used, the data represented in the 2D $H_2$ plot for both 2DOF and 5DOF were fully within the Nyquist regions of $[0-200]Hz$. Therefore, the presented differences between the plots were likely a result of the limitation of the algorithm rather than issues with aliasing misrepresenting the FRFs of the system since the 0% noise predictions were very strong already; however, Nyquist regions for $n>1$ are more complicated.

## $H_3$ kernel prediction

**Fig. 19:**
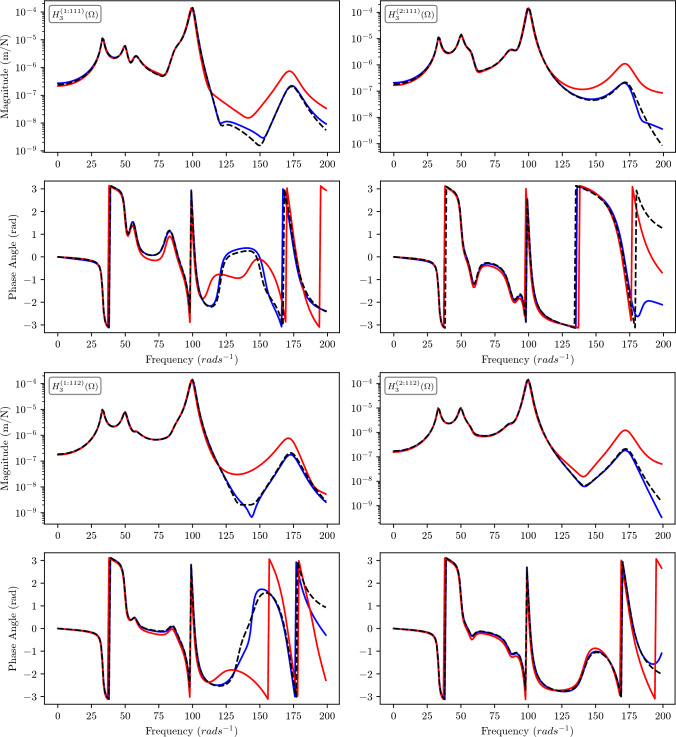
Comparison of $H_3$ kernel predictions for a 2DOF system measured at mass one and two and excited by three unique frequencies at point one for the top four figures, then two unique frequencies at point one and a further unique frequency at point two for the bottom four figures

The probing method for the $H_3$ kernel was applied to the 2DOF system, presented in Fig. 19. This much more complicated extraction required more computational power to recover the 3-dimensional matrices representing the kernel. Therefore, only the diagonal of the 2DOF oscillator case kernels was used in presenting the predictions, also indicating some discrepancies from probing a network at this order. The forcing chosen gave probing measurements at Points One and Two, forced with three different harmonics at Point One. The prediction accuracy was good, with the clear shape of magnitude and phase captured by the neural network. This prediction, however, had noticeable discrepancies when compared with $H_1$ and $H_2$. This effect could be a result of truncation of the Volterra series and therefore the bulk of the excess kernels were possibly being absorbed into $H_3$. Also, $H_3$ is made up of $H_1$ and $H_2$ predictions, therefore, any errors accumulated in the two previous predictions is carried forward, likely leading to these additional discrepancies. Regardless, the predictions for the $H_3$ kernel of the 2DOF system were good as the important data related to peak location, magnitude and phase could still be adopted for further analysis (Fig. 19).

**Table 1:** Test NMSE and MAE comparison for 2DOF and 5DOF cases

	2DOF		5DOF	
	0%	5%	0%	5%
NMSE	0.01867	1.30093	0.09914	4.87546
MAE	0.008697	0.07003	0.01996	0.16538
Avg Comp Time	327.7 s		358.9 s	

## Discussion

By observing the recovered kernels for both the 2DOF and 5DOF examples, there is clearly a very good prediction accuracy, where the shape of the recovered kernels clearly follows the theoretical kernel for both noise cases up to $3^{rd}$ order. Most notably the 2DOF example showed very robust agreements for all cases. Because of the simpler system set-up and less kernel errors being propagated forward (as will be referenced later), the NARX NN was able to learn the system better and therefore produce more accurate kernel visualisation using the trained weights. Since the NARX method is not an exact approximation of the system, error can be present in the predictions as seen in Table 1.

The more NMSE error in the 5DOF case is noticeably larger than the 2DOF case for both noise scenarios. The results suggested that because of the increase in the number of input channels, the error in forward propagation is larger, leading to a worse (but still good), agreement considering the noise present. Referencing Parseval’s theorem, the error observed in the time domain is comparable in the frequency domain and therefore the chosen network can suitably use the recovered weights to predict the system well in both cases, supported by comparison with the true HFRFs. The discrepancies help to highlight that the information contained in the kernel prediction is most influential at frequencies less than $$ 100 rads^{-1}$$ since the HFRF predictions were very close for all cases in that range. The linear kernel is, as expected, most reliably recovered, highlighting its contribution to the output; however, truncating the activation function to order 3 and manipulating $$H_1(\varOmega )$$ using higher unseen harmonics to generate $$H_1(2\varOmega )$$ and $$H_1(3\varOmega )$$ within the desired frequency range could have led to some of the differences observed in the nonlinear kernel predictions. This problem could be resolved by either increasing the activation function order or applying the chosen harmonic probing algorithm to other machine-learning approaches such as a Gaussian Processes, to see if the predictions difficulties are consistent. It is evident though, that the kernel prediction accuracy and MPO accuracy are strong indications of a well-trained network.

Noise evidently affects prediction accuracy and increasing the magnitude of noise would make HFRF recovery much more difficult for systems with many degrees of freedom. However, training the systems using different model structures can help indicate the true kernel structure or a poorly trained model. The prediction of the linear kernel directly affects the structure of the higher-order kernels. Referencing the recovery equations in Appendix C, the nonlinear kernels use the recovered lower-order kernel equations to be predicted themselves alongside the same weights and the relevant order’s activation function coefficient. Therefore, the higher-order kernels will always be less accurate than the lower-order kernels, suggesting more effort should be focussed on understanding the underlying linearity to avoid possible error propagation through the higher-order kernels. Increasing the number of orders used to represent the Taylor expansion of Tanh should also be prioritised to improve kernel predictions. The radius of convergence makes the approach difficult when higher-orders are included because of the approximation of Tanh outside the radius being more abstract. Therefore, methods of constraining the weights to control this should be explored to improve consistent kernel recovery that is more accurate. If one was to extend this research, work should be focussed on constraining the NARX NN to construct the correct kernel structures from multiple consistent kernel recoveries. Such as using different initialised weights or training on limited sections of available data. Altering the structure of the network–such as the number of time-lags, weights initialisations, Taylor series truncation, and other hyperparameters–could all help recover an average true kernel structure. The system was not too sensitive to hyperparameter alterations and the derived harmonic-probing algorithms are flexible to allow HFRF construction for different hidden sizes and lag choices. The results in this case, however, show clear structure for all three levels of nonlinearity recovered compared with theory and therefore alongside the MPO error, one can be confident in using the recovered kernels to inform the user of the nonlinearity within the system.

This development would allow a natural extension toward implementation for similar nonlinear dynamical models such as GP-NARX. However, in the MIMO setting these approaches face practical limitations, particularly in terms of scalability and the complexity of defining suitable kernel structures across multiple inputs and outputs. One does, however, intend to focus the work in this direction to provide a currently unavailable comparison for the predicted MDOF HFRFs. This would allow a better understanding towards the efficiency of the neural network predictions, eventually presenting work surrounding interpreting and making useful decisions from these HFRFs harnessing the benefits of these contrasting models. The computational burden and model design become increasingly restrictive as system dimensionality grows, constraints that motivated the choice of neural network–based formulations in this work, which offer greater flexibility and scalability for MIMO Volterra kernel recovery while still capturing complex nonlinear dynamics.

## Conclusions

It has been shown that the method of harmonic probing of neural networks for SDOF models can be extended to accommodate MIMO systems and predict higher-order kernels in the frequency domain. The method was adapted to accommodate systems of *n*-degrees-of-freedom, as well as forecast unseen test data to a high accuracy, even with reasonably large noise pollution. This research has shown how the difficult derivation of these higher-order kernels from physical models could be bypassed using HFRF relationships to probe the neural networks equation and recover the relevant HFRF based on the weights and biases. Thus, neural networks can be trained to recover and present good nonlinear kernel transform predictions in the frequency-domain for time-invariant nonlinear MIMO systems (see Fig. 4), promoting the potential implementation of the algorithm on physical time-invariant systems. As a result of this research, one has the possibility of recovering HFRFs from network weights as an additional way to judge whether a neural network has been suitably trained, as well as giving important information about the true levels of nonlinearity within a system. This additional information promotes the adoption of harmonic probing to recover MIMO HFRFs in other machine-learning architectures and encourages taking advantage of the specific strengths of each to improve the understanding of complicated nonlinear dynamic systems via the Volterra series.

**Fig. 20:**
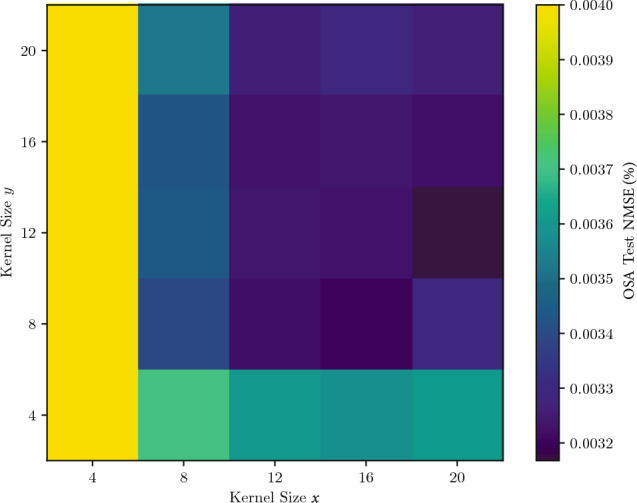
OSA Grid Search on the 2DOF system to show the sensitivity of hyperparameter selection

**Fig. 21:**
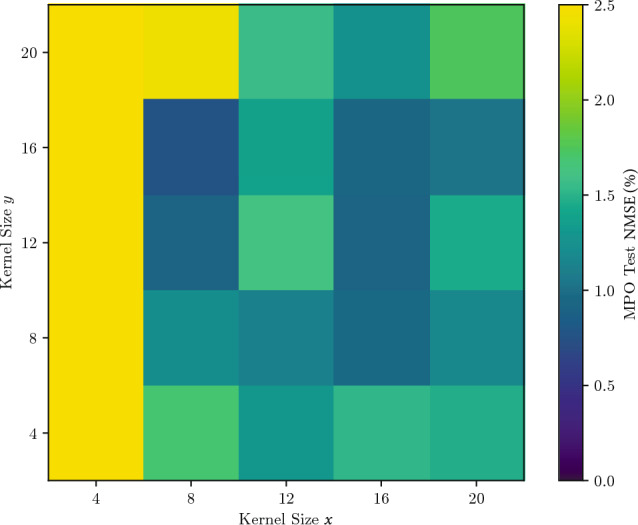
MPO Grid Search on the 2DOF system to show the sensitivity of hyperparameter selection

**Fig. 22:**
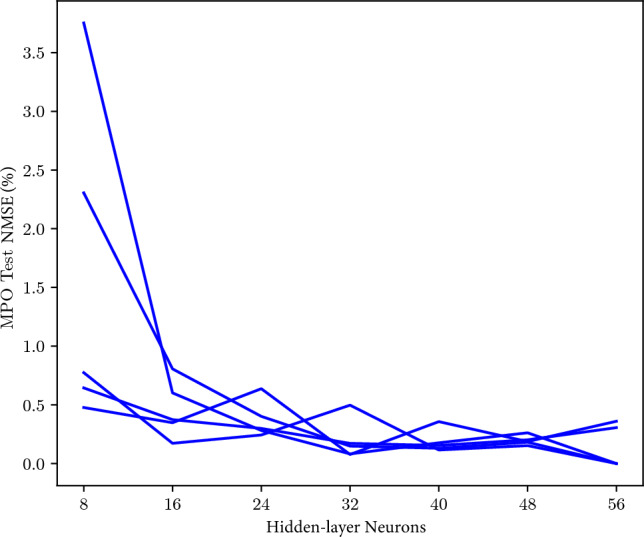
Hidden-layer neuron size search to visualise the effect on the MPO NMSE using five different weight initialisations ($k_x = k_y = 10$)

**Fig. 23:**
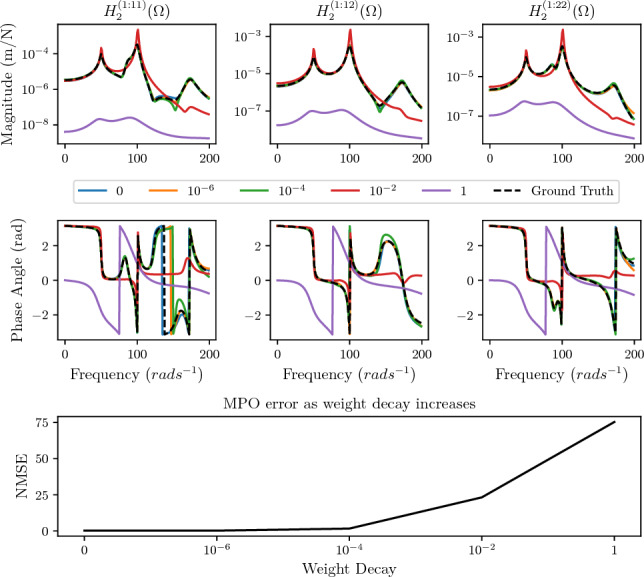
Subplots showing the effect of increasing the magnitude of weight decay on the recovered $H_2$ HFRFs, as well as on the MPO test NMSE ($20-32-2$ NN structure)

## Data Availability

Information required to simulate the data used in this study is provided within the manuscript.

## Hyperparameter optimisations

The kernel size for exogenous and autoregressive input channels were trained for with a range of hidden neurons and data with $1\%$ noise pollution. The chosen kernel sizes were then used to find the best number of hidden neurons.

See Figs. 20, 21, 22, 23.

After observing the hyperparameter search, kernel sizes for both autoregressive and exogenous input channels were kept the same. This had negligible effect on the accuracy and symmetry in the kernel structure simplifies the probing process. Varying the hidden-layer neurons had a noticeable effect, where a larger hidden layer provided better predictions. The results plateaued around 32 neurons and therefore this was used in the network to reduce overfitting.

In the development of the model, a sensitivity analysis was conducted on the weight decay coefficient for the Adam optimizer. While weight decay is a standard technique for regularization in many deep learning contexts, our results (determined from the average of three weight initialisations) indicated a negative correlation between the magnitude of weight decay and model performance. Early stopping and reducing the learning rate at points of convergence was sufficient in preventing overfitting without the detrimental effects observed with Adam-coupled weight decay.

## Theoretical kernel recovery derivation

To assist in understanding how the harmonic probing approach works, a more detailed worked example has been completed. This is a more in-depth extension of the 2DOF system presented earlier in the paper to fully understand the process. For the 2DOF system presented in Fig. 3, the following equations of motion can be derived,

56 $$\begin{aligned}  &   m\ddot{y}^{(1)} + 2c\dot{y}^{(1)} - c\dot{y}^{(2)} + 2ky^{(1)} - ky^{(2)} \nonumber \\  &   \quad + k_2{y^{(1)}}^2 + k_3{y^{(1)}}^3 = x^{(1)}(t) \end{aligned}$$

57 $$\begin{aligned}  &   m\ddot{y}^{(2)} + 2c\dot{y}^{(2)} - c\dot{y}^{(1)} + 2ky^{(2)} - ky^{(1)} \nonumber \\  &   \quad + k_2{y^{(2)}}^2 + k_3{y^{(2)}}^3 = x^{(2)}(t) \end{aligned}$$

For the first-order kernel, the probing inputs required are represented by a single harmonic at *p* and a lack of input at *q*,

58 $$\begin{aligned} x^{(p)}(t)= &   e^{i\varOmega t} \end{aligned}$$

59 $$\begin{aligned} x^{(q)}= &   0 \end{aligned}$$

giving output contributions only accounting for input *p*,

60 $$\begin{aligned} y^{(p)}(t)= &   H_1^{(p:p)}(\varOmega )e^{i\varOmega t} \end{aligned}$$

61 $$\begin{aligned} y^{(q)}(t)= &   H_1^{(q:p)}(\varOmega )e^{i\varOmega t} \end{aligned}$$

Inserting these probing equations into Eqs. (56) and (57) for the 2DOF system, four separate equations can be derived.

For excitation at point 1,

62 $$\begin{aligned}&-m\varOmega ^2 H_1^{(1:1)}(\varOmega )e^{i\varOmega t} + 2i\varOmega c H_1^{(1:1)}(\varOmega )e^{i\varOmega t} \nonumber \\&\quad - ic H_1^{(2:1)}(\varOmega )e^{i\varOmega t}\nonumber \\&+ 2kH_1^{(1:1)}(\varOmega )e^{i\varOmega t} - kH_1^{(2:1)}(\varOmega )e^{i\varOmega t} \nonumber \\&\quad + k_2 {H_1^{(1:1)}(\varOmega )}^2 e^{2i\varOmega t}\nonumber \\&+ k_3 {H_1^{(1:1)}(\varOmega )}^3 e^{3i\varOmega t} = e^{i\varOmega t} \end{aligned}$$

63 $$\begin{aligned}&-m\varOmega ^2 H_1^{(2:1)}(\varOmega )e^{i\varOmega t} + 2i\varOmega c H_1^{(2:1)}(\varOmega )e^{i\varOmega t} \nonumber \\&\quad - ic H_1^{(1:1)}(\varOmega )e^{i\varOmega t}\nonumber \\&+ 2kH_1^{(2:1)}(\varOmega )e^{i\varOmega t} - kH_1^{(1:1)}(\varOmega )e^{i\varOmega t} \nonumber \\&\quad + k_2 {H_1^{(2:1)}(\varOmega )}^2 e^{2i\varOmega t}\nonumber \\&+ k_3 {H_1^{(2:1)}(\varOmega )}^3 e^{3i\varOmega t} = 0 \end{aligned}$$

For excitation at point 2,

64 $$\begin{aligned}&-m\varOmega ^2 H_1^{(1:2)}(\varOmega )e^{i\varOmega t} + 2i\varOmega c H_1^{(1:2)}(\varOmega )e^{i\varOmega t} \nonumber \\&\quad - ic H_1^{(2:2)}(\varOmega )e^{i\varOmega t}\nonumber \\&+ 2kH_1^{(1:2)}(\varOmega )e^{i\varOmega t} - kH_1^{(2:2)}(\varOmega )e^{i\varOmega t} \nonumber \\&\quad + k_2 {H_1^{(1:2)}(\varOmega )}^2 e^{2i\varOmega t}\nonumber \\&+ k_3 {H_1^{(1:2)}(\varOmega )}^3 e^{3i\varOmega t} = 0 \end{aligned}$$

65 $$\begin{aligned}&-m\varOmega ^2 H_1^{(2:2)}(\varOmega )e^{i\varOmega t} + 2i\varOmega c H_1^{(2:2)}(\varOmega )e^{i\varOmega t} \nonumber \\&\quad - ic H_1^{(1:2)}(\varOmega )e^{i\varOmega t}\nonumber \\&+ 2kH_1^{(2:2)}(\varOmega )e^{i\varOmega t} - kH_1^{(1:2)}(\varOmega )e^{i\varOmega t} \nonumber \\&\quad + k_2 {H_1^{(2:2)}(\varOmega )}^2 e^{2i\varOmega t}\nonumber \\  &+ k_3 {H_1^{(2:2)}(\varOmega )}^3 e^{3i\varOmega t} = e^{i\varOmega t} \end{aligned}$$

After equating the coefficients of $e^{i\varOmega t}$, the nonlinear terms are removed, leaving four equations and four unknowns (each unique $H_1$) which can be solved using matrix algebra.

66 $$\begin{aligned} \mathbf {H_1}^{(2)}(\varOmega )&= \begin{bmatrix} H_1^{(1:1)}(\varOmega ) &  H_1^{(1:2)}(\varOmega ) \\ H_1^{(2:1)}(\varOmega ) &  H_1^{(2:2)}(\varOmega ) \\ \end{bmatrix}\nonumber \\&=\begin{bmatrix} \begin{array}{c}-m\varOmega ^2 + 2k +2ci\varOmega \end{array} &  -ci\varOmega - k \\ -ci\varOmega - k &  \begin{array}{c}-m\varOmega ^2 + 2k + 2ci\varOmega \end{array} \\ \end{bmatrix}^{-1} \end{aligned}$$

Different probing equations are required for the second order kernel, simplified down to two separate scenarios. Here, two independent harmonics excite the system at a single point together or two points separately.

67 $$\begin{aligned} x^{(p)}(t)= &   e^{i\varOmega _1 t} + e^{i\varOmega _2 t} \end{aligned}$$

68 $$\begin{aligned} x^{(q)}= &   0 \end{aligned}$$

or

69 $$\begin{aligned} x^{(p)}(t)= &   e^{i\varOmega _1 t} \end{aligned}$$

70 $$\begin{aligned} x^{(q)}= &   e^{i\varOmega _2 t} \end{aligned}$$

giving output contributions respectively of,

71 $$\begin{aligned} y^{(p)}(t)&= H_1^{(p:p)}(\varOmega _1)e^{i\varOmega _1 t} + H_1^{(p:p)}(\varOmega _2)e^{i\varOmega _2 t}\nonumber \\&+ H_2^{(p:pp)}(\varOmega _1 + \varOmega _2)e^{i(\varOmega _1 +\varOmega _2) t} \end{aligned}$$

72 $$\begin{aligned} y^{(q)}(t)&= H_1^{(q:p)}(\varOmega _1)e^{i\varOmega _1 t} + H_1^{(q:p)}(\varOmega _2)e^{i\varOmega _2 t}\nonumber \\  &+ H_2^{(q:pp)}(\varOmega _1 + \varOmega _2)e^{i(\varOmega _1 +\varOmega _2) t} \end{aligned}$$

73 $$\begin{aligned} y^{(p)}(t)&= H_1^{(p:p)}(\varOmega _1)e^{i\varOmega _1 t} + H_1^{(p:q)}(\varOmega _2)e^{i\varOmega _2 t}\nonumber \\  &+ H_2^{(p:pq)}(\varOmega _1 + \varOmega _2)e^{i(\varOmega _1 +\varOmega _2) t} \end{aligned}$$

74 $$\begin{aligned} y^{(q)}(t)&= H_1^{(q:p)}(\varOmega _1)e^{i\varOmega _1 t} + H_1^{(q:q)}(\varOmega _2)e^{i\varOmega _2 t}\nonumber \\  &+ H_2^{(q:pq)}(\varOmega _1 + \varOmega _2)e^{i(\varOmega _1 +\varOmega _2) t} \end{aligned}$$

Evidently for Eqs. (73) and (74), swapping the excitation points of the harmonics ($\varOmega _1$ and $\varOmega _2$) would give the same result ($H_2^{(p:pq)} = H_2^{(p:qp)}$). Therefore, the total amount of second order kernels to be determined is greatly reduced through symmetry.

Probing (56) and (57) with these new equations (using symmetry and equating coefficients of $e^{i(\varOmega _1 + \varOmega _2)}$ for excitations at point 1) gives,

75 $$\begin{aligned}&-m(\varOmega _1 + \varOmega _2)^2 H_2^{(1:11)}(\varOmega _1,\varOmega _2) \nonumber \\&\quad + 2ic(\varOmega _1 + \varOmega _2) H_2^{(1:11)}(\varOmega _1,\varOmega _2)\nonumber \\&- ic(\varOmega _1 + \varOmega _2)H_2^{(2:11)}(\varOmega _1,\varOmega _2) + 2kH_2^{(1:11)}(\varOmega _1,\varOmega _2)\nonumber \\&- kH_2^{(2:11)}(\varOmega _1,\varOmega _2) + k_2 H_1^{(1:1)}(\varOmega _1)H_1^{(1:1)}(\varOmega _2) = 0 \end{aligned}$$

76 $$\begin{aligned}&-m(\varOmega _1 + \varOmega _2)^2 H_2^{(2:11)}(\varOmega _1,\varOmega _2) \nonumber \\&\quad + 2ic(\varOmega _1 + \varOmega _2) H_2^{(2:11)}(\varOmega _1,\varOmega _2)\nonumber \\&\quad - ic(\varOmega _1 + \varOmega _2)H_2^{(1:11)}(\varOmega _1,\varOmega _2) + 2kH_2^{(2:11)}(\varOmega _1,\varOmega _2)\nonumber \\&- kH_2^{(1:11)}(\varOmega _1,\varOmega _2) + k_2 H_1^{(2:1)}(\varOmega _1)H_1^{(2:1)}(\varOmega _2) = 0 \end{aligned}$$

For excitation at point 1 and 2 this gives,

77 $$\begin{aligned}&-m(\varOmega _1 + \varOmega _2)^2 H_2^{(1:12)}(\varOmega _1,\varOmega _2) \nonumber \\&\quad + 2ic(\varOmega _1 + \varOmega _2) H_2^{(1:12)}(\varOmega _1,\varOmega _2)\nonumber \\&- ic(\varOmega _1 + \varOmega _2)H_2^{(2:12)}(\varOmega _1,\varOmega _2) + 2kH_2^{(1:12)}(\varOmega _1,\varOmega _2)\nonumber \\&- kH_2^{(2:12)}(\varOmega _1,\varOmega _2) + k_2 H_1^{(1:1)}(\varOmega _1)H_1^{(1:2)}(\varOmega _2) = 0 \end{aligned}$$

78 $$\begin{aligned}&-m(\varOmega _1 + \varOmega _2)^2 H_2^{(2:12)}(\varOmega _1,\varOmega _2) \nonumber \\&\quad + 2ic(\varOmega _1 + \varOmega _2) H_2^{(2:12)}(\varOmega _1,\varOmega _2)\nonumber \\&- ic(\varOmega _1 + \varOmega _2)H_2^{(1:12)}(\varOmega _1,\varOmega _2) + 2kH_2^{(2:12)}(\varOmega _1,\varOmega _2)\nonumber \\&- kH_2^{(1:12)}(\varOmega _1,\varOmega _2) + k_2 H_1^{(2:1)}(\varOmega _1)H_1^{(2:2)}(\varOmega _2) = 0 \end{aligned}$$

For excitations at point 2 this gives,

79 $$\begin{aligned}&-m(\varOmega _1 + \varOmega _2)^2 H_2^{(1:22)}(\varOmega _1,\varOmega _2) \nonumber \\&\quad + 2ic(\varOmega _1 + \varOmega _2) H_2^{(1:22)}(\varOmega _1,\varOmega _2)\nonumber \\&- ic(\varOmega _1 + \varOmega _2)H_2^{(2:22)}(\varOmega _1,\varOmega _2) + 2kH_2^{(1:22)}(\varOmega _1,\varOmega _2)\nonumber \\&- kH_2^{(2:22)}(\varOmega _1,\varOmega _2) + k_2 H_1^{(1:2)}(\varOmega _1)H_1^{(1:2)}(\varOmega _2) = 0 \end{aligned}$$

80 $$\begin{aligned}&-m(\varOmega _1 + \varOmega _2)^2 H_2^{(2:22)}(\varOmega _1,\varOmega _2) \nonumber \\&\quad + 2ic(\varOmega _1 + \varOmega _2) H_2^{(2:22)}(\varOmega _1,\varOmega _2)\nonumber \\&- ic(\varOmega _1 + \varOmega _2)H_2^{(1:22)}(\varOmega _1,\varOmega _2) + 2kH_2^{(2:22)}(\varOmega _1,\varOmega _2)\nonumber \\&- kH_2^{(1:22)}(\varOmega _1,\varOmega _2) + k_2 H_1^{(2:2)}(\varOmega _1)H_1^{(2:2)}(\varOmega _2) = 0 \end{aligned}$$

Noticeably for each of the three different input combinations above, the $H_2$ terms to be determined are exclusively shared within these three instances. Therefore, for each instance there are two equations and two unknowns. Meaning that solving for excitations at point one alone to recover $H_2$, is just like solving for $H_1$ which leads to,

81 $$\begin{aligned}&\begin{bmatrix} H_2^{(1:11)}(\varOmega _1, \varOmega _2) \\ H_2^{(2:11)}(\varOmega _1, \varOmega _2) \\ \end{bmatrix} =\begin{bmatrix} \begin{array}{c}-m(\varOmega _1 + \varOmega _2)^2 + 2k +2ci(\varOmega _1 + \varOmega _2)\end{array} &  -ci(\varOmega _1 + \varOmega _2) - k \\ -ci(\varOmega _1 + \varOmega _2) - k &  \begin{array}{c}-m(\varOmega _1 + \varOmega _2)^2 + 2k + 2ci(\varOmega _1 + \varOmega _2)\end{array} \\ \end{bmatrix}^{-1}\nonumber \\&\begin{bmatrix} -k_2 H_1^{(1:1)}(\varOmega _1) H_1^{(1:1)}(\varOmega _2) \\ -k_2 H_1^{(2:1)}(\varOmega _1) H_1^{(2:1)}(\varOmega _2) \\ \end{bmatrix} \end{aligned}$$

which can be simplified down to,

82 $$\begin{aligned}  &   \begin{bmatrix} H_2^{(1:11)}(\varOmega _1, \varOmega _2) \\ H_2^{(2:11)}(\varOmega _1, \varOmega _2) \\ \end{bmatrix} =H_1^{(2)}(\varOmega _1 + \varOmega _2)\nonumber \\  &   \quad \begin{bmatrix} -k_2 H_1^{(1:1)}(\varOmega _1) H_1^{(1:1)}(\varOmega _2) \\ -k_2 H_1^{(2:1)}(\varOmega _1) H_1^{(2:1)}(\varOmega _2) \\ \end{bmatrix}. \end{aligned}$$

This technique works for the specific nonlinear mass-spring-damper systems presented in the paper and can be adjusted to whichever system is relevant. However, this adjustment can present issues as much more complicated systems will require much more tedious maths to recover HFRFs from MDOF equations of motion. Therefore, the harmonically probed neural network method, using similar probing techniques to this theoretical equation of motion HFRF recovery method, helps to find the correct HFRFs much easier.

## Neural network based kernel recovery derivations

### $H_1$ kernel derivation (*n* orders)

The measured data is discrete and therefore, the single hidden layer NARX neural network equation for ndof system recovery is presented below,

83 $$\begin{aligned} y^{(p)}_t&= \sum _{i=0}^{\infty } \sum _{j=0}^{n_h} \frac{w_j^{(p)}{\tanh ^{(i)}(b_j)}}{i!}\Big (\sum _{m = 0}^{n_x-1}u^{(1)}_{jm}x^{(1)}_{t-m} + \cdots \end{aligned}$$

84 $$\begin{aligned}&+ \sum _{m=0}^{n_x-1}u^{(n)}_{jm}x^{(n)}_{t-m} + \sum _{k = 1}^{n_y}v^{(1)}_{jk}y^{(1)}_{t-k} + \cdots + \sum _{k=1}^{n_y}v^{(n)}_{jk}y^{(n)}_{t-k} \Big )^i. \end{aligned}$$

This requires probing with the following discretised input and output representations,

85 $$\begin{aligned} x^{(p)}_{t-m}= &   e^{i\varOmega (t-m\delta t)}, \end{aligned}$$

86 $$\begin{aligned} y^{(p)}_{t-k}= &   H_1^{(p:p)}(\varOmega )e^{i\varOmega (t-k\delta t)}, \end{aligned}$$

87 $$\begin{aligned} y^{(q)}_{t-k}= &   H_1^{(q:p)}(\varOmega )e^{i\varOmega (t-k\delta t)}. \end{aligned}$$

For a simplified example, $n = 2$ was chosen, and when equating coefficients of $e^{i\varOmega t}$, this gives four probed equations like in the theoretical case.

88 $$\begin{aligned} H_1^{(1:1)}&= \sum _{j=0}^{n_h} w_j^{(1)} \tanh (b_j) \Bigg ( \sum _{m=0}^{n_x-1} u^{(1)}_{jm} e^{-i\varOmega m\delta t} + \sum _{m=0}^{n_x-1} u^{(2)}_{jm} e^{-i\varOmega m\delta t}\nonumber \\&+ \sum _{k=1}^{n_y} v^{(1)}_{jk} H_1^{(1:1)}(\varOmega ) e^{-i\varOmega k\delta t} + \sum _{k=1}^{n_y} v^{(2)}_{jk} H_1^{(2:1)}(\varOmega ) e^{-i\varOmega k\delta t} \Bigg ) \end{aligned}$$

89 $$\begin{aligned} H_1^{(2:1)}&= \sum _{j=0}^{n_h} w_j^{(1)} \tanh (b_j) \Bigg ( \sum _{m=0}^{n_x-1} u^{(1)}_{jm} e^{-i\varOmega m\delta t} + \sum _{m=0}^{n_x-1} u^{(2)}_{jm} e^{-i\varOmega m\delta t}\nonumber \\&+ \sum _{k=1}^{n_y} v^{(1)}_{jk} H_1^{(1:1)}(\varOmega ) e^{-i\varOmega k\delta t} + \sum _{k=1}^{n_y} v^{(2)}_{jk} H_1^{(2:1)}(\varOmega ) e^{-i\varOmega k\delta t} \Bigg ) \end{aligned}$$

90 $$\begin{aligned} H_1^{(1:2)}&= \sum _{j=0}^{n_h} w_j^{(1)} \tanh (b_j) \Bigg ( \sum _{m=0}^{n_x-1} u^{(1)}_{jm} e^{-i\varOmega m\delta t} + \sum _{m=0}^{n_x-1} u^{(2)}_{jm} e^{-i\varOmega m\delta t}\nonumber \\&+ \sum _{k=1}^{n_y} v^{(1)}_{jk} H_1^{(1:2)}(\varOmega ) e^{-i\varOmega k\delta t} + \sum _{k=1}^{n_y} v^{(2)}_{jk} H_1^{(2:2)}(\varOmega ) e^{-i\varOmega k\delta t} \Bigg ) \end{aligned}$$

91 $$\begin{aligned} H_1^{(2:2)}&= \sum _{j=0}^{n_h} w_j^{(1)} \tanh (b_j) \Bigg ( \sum _{m=0}^{n_x-1} u^{(1)}_{jm} e^{-i\varOmega m\delta t} + \sum _{m=0}^{n_x-1} u^{(2)}_{jm} e^{-i\varOmega m\delta t}\nonumber \\&+ \sum _{k=1}^{n_y} v^{(1)}_{jk} H_1^{(1:2)}(\varOmega ) e^{-i\varOmega k\delta t} + \sum _{k=1}^{n_y} v^{(2)}_{jk} H_1^{(2:2)}(\varOmega ) e^{-i\varOmega k\delta t} \Bigg ) \end{aligned}$$

Solving for $H_1$, there are four equations and four unknowns which leads to,

92 $$\begin{aligned}&\begin{bmatrix} 1 - \sum _{j=1}^{nh}w_{j}^{(1)}\tanh ^{(1)}(b_j)\sum _{k=1}^{ny}v_{jk}^{(1)}e^{-ik\delta t\varOmega } &  -\sum _{j=1}^{nh}w_{j}^{(1)}\tanh ^{(1)}(b_j)\sum _{k=1}^{ny}v_{jk}^{(2)}e^{-ik\delta t \varOmega }\\ -\sum _{j=1}^{nh}w_{j}^{(2)}\tanh ^{(1)}(b_j)\sum _{k=1}^{ny}v_{jk}^{(1)}e^{-ik\delta t \varOmega } &  1 - \sum _{j=1}^{nh}w_{j}^{(2)}\tanh ^{(1)}(b_j)\sum _{k=1}^{ny}v_{jk}^{(2)}e^{-ik\delta t\varOmega } \end{bmatrix}\nonumber \\&\begin{bmatrix} H_1^{(1:1)}(\varOmega ) &  H_1^{(1:2)}(\varOmega ) \\ H_1^{(2:1)}(\varOmega ) &  H_1^{(2:2)}(\varOmega ) \\ \end{bmatrix}\nonumber \\&= \begin{bmatrix} \sum _{j=1}^{nh}w_{j}^{(1)}\tanh ^{(1)}(b_j)\sum _{m=0}^{n_x-1}u_{jm}^{(1)}e^{-im\delta t\varOmega } &  \sum _{j=1}^{nh}w_{j}^{(1)}\tanh ^{(1)}(b_j)\sum _{m=0}^{n_x-1}u_{jm}^{(2)}e^{-im\delta t\varOmega }\\ \sum _{j=1}^{nh}w_{j}^{(2)}\tanh ^{(1)}(b_j)\sum _{m=0}^{n_x-1}u_{jm}^{(1)}e^{-im\delta t\varOmega } &  \sum _{j=1}^{nh}w_{j}^{(2)}\tanh ^{(1)}(b_j)\sum _{m=0}^{n_x-1}u_{jm}^{(2)}e^{-im\delta t\varOmega } \end{bmatrix}. \end{aligned}$$

The previous 2DOF equation can intuitively be extended to *n* orders by observing the trends in the constructed matrices,

93 $$\begin{aligned}  &   \begin{aligned}&\begin{bmatrix} 1 - \sum _{j=1}^{nh}w_{j}^{(1)}\tanh ^{(1)}(b_j)\sum _{k=1}^{ny}v_{jk}^{(1)}e^{-ik\delta t\varOmega } &  -\sum _{j=1}^{nh}w_{j}^{(1)}\tanh ^{(1)}(b_j)\sum _{k=1}^{ny}v_{jk}^{(2)}e^{-ik\delta t \varOmega } & \cdots & -\sum _{j=1}^{nh}w_{j}^{(1)}\tanh ^{(1)}(b_j)\sum _{k=1}^{ny}v_{jk}^{(n)}e^{-ik\delta t \varOmega }\\ -\sum _{j=1}^{nh}w_{j}^{(2)}\tanh ^{(1)}(b_j)\sum _{k=1}^{ny}v_{jk}^{(1)}e^{-ik\delta t \varOmega } &  1 - \sum _{j=1}^{nh}w_{j}^{(2)}\tanh ^{(1)}(b_j)\sum _{k=1}^{ny}v_{jk}^{(2)}e^{-ik\delta t\varOmega } & \cdots & -\sum _{j=1}^{nh}w_{j}^{(2)}\tanh ^{(1)}(b_j)\sum _{k=1}^{ny}v_{jk}^{(n)}e^{-ik\delta t \varOmega }\\ \vdots &  \vdots &  \ddots &  \vdots \\ -\sum _{j=1}^{nh}w_{j}^{(n)}\tanh ^{(1)}(b_j)\sum _{k=1}^{ny}v_{jk}^{(1)}e^{-ik\delta t \varOmega } &  - \sum _{j=1}^{nh}w_{j}^{(n)}\tanh ^{(1)}(b_j)\sum _{k=1}^{ny}v_{jk}^{(2)}e^{-ik\delta t\varOmega } & \cdots &  1 -\sum _{j=1}^{nh}w_{j}^{(n)}\tanh ^{(1)}(b_j)\sum _{k=1}^{ny}v_{jk}^{(n)}e^{-ik\delta t \varOmega }\\ \end{bmatrix}\\&\begin{bmatrix} H_1^{(1:1)}(\varOmega ) &  H_1^{(1:2)}(\varOmega ) &  \cdots &  H_1^{(1:n)}(\varOmega ) \\ H_1^{(2:1)}(\varOmega ) &  H_1^{(2:2)}(\varOmega ) &  \cdots &  H_1^{(2:n)}(\varOmega ) \\ \vdots &  \vdots &  \ddots &  \vdots \\ H_1^{(n:1)}(\varOmega ) &  H_1^{(n:2)}(\varOmega ) &  \cdots &  H_1^{(n:n)}(\varOmega ) \end{bmatrix} \end{aligned} \nonumber \\  &   \begin{aligned}&= \begin{bmatrix} \sum _{j=1}^{nh}w_{j}^{(1)}\tanh ^{(1)}(b_j)\sum _{m=0}^{n_x-1}u_{jm}^{(1)}e^{-im\delta t\varOmega } &  \sum _{j=1}^{nh}w_{j}^{(1)}\tanh ^{(1)}(b_j)\sum _{m=0}^{n_x-1}u_{jm}^{(2)}e^{-im\delta t\varOmega } & \cdots &  \sum _{j=1}^{nh}w_{j}^{(1)}\tanh ^{(1)}(b_j)\sum _{m=0}^{n_x-1}u_{jm}^{(n)}e^{-im\delta t\varOmega }\\ \sum _{j=1}^{nh}w_{j}^{(2)}\tanh ^{(1)}(b_j)\sum _{m=0}^{n_x-1}u_{jm}^{(1)}e^{-im\delta t\varOmega } &  \sum _{j=1}^{nh}w_{j}^{(2)}\tanh ^{(1)}(b_j)\sum _{m=0}^{n_x-1}u_{jm}^{(2)}e^{-im\delta t\varOmega } & \cdots &  \sum _{j=1}^{nh}w_{j}^{(2)}\tanh ^{(1)}(b_j)\sum _{m=0}^{n_x-1}u_{jm}^{(n)}e^{-im\delta t\varOmega }\\ \vdots &  \vdots &  \ddots &  \vdots \\ \sum _{j=1}^{nh}w_{j}^{(n)}\tanh ^{(1)}(b_j)\sum _{m=0}^{n_x-1}u_{jm}^{(1)}e^{-im\delta t\varOmega } &  \sum _{j=1}^{nh}w_{j}^{(n)}\tanh ^{(1)}(b_j)\sum _{m=0}^{n_x-1}u_{jm}^{(2)}e^{-im\delta t\varOmega } & \cdots &  \sum _{j=1}^{nh}w_{j}^{(n)}\tanh ^{(1)}(b_j)\sum _{m=0}^{n_x-1}u_{jm}^{(n)}e^{-im\delta t\varOmega } \end{bmatrix} \end{aligned} \nonumber \\ \end{aligned}$$

### $H_2$ kernel derivation (*n* orders)

This methodology is extended to $H_2$ by transforming the corresponding harmonic probing equations into discrete time. By equating the coefficients of the relevant harmonics, the HFRFs can be isolated and recovered. The resulting mathematical structure is analogous to that of $H_1$; however, the inclusion of additional harmonics means accounting for multiple combinations of lower-order kernels that contribute to the overall response is required.

94 $$\begin{aligned}&\begin{aligned}&\begin{bmatrix} 1-\sum _{j=1}^{nh}w_{j}^{(1)}\tanh ^{(1)}(b_j)\sum _{k=1}^{n_y}v_{jk}^{(1)}e^{-i(\varOmega _1 +\varOmega _2)k\delta t} &  -\sum _{j=1}^{nh}w_{j}^{(1)}\tanh ^{(1)}(b_j)\sum _{k=1}^{n_y}v_{jk}^{(2)}e^{-i(\varOmega _1 +\varOmega _2)k\delta t} & \cdots &  -\sum _{j=1}^{nh}w_{j}^{(1)}\tanh ^{(1)}(b_j)\sum _{k=1}^{n_y}v_{jk}^{(n)}e^{-i(\varOmega _1 +\varOmega _2)k\delta t}\\ -\sum _{j=1}^{nh}w_{j}^{(2)}\tanh ^{(1)}(b_j)\sum _{k=1}^{n_y}v_{jk}^{(1)}e^{-i(\varOmega _1 +\varOmega _2)k\delta t} &  1-\sum _{j=1}^{nh}w_{j}^{(2)}\tanh ^{(1)}(b_j)\sum _{k=1}^{n_y}v_{jk}^{(2)}e^{-i(\varOmega _1 +\varOmega _2)k\delta t} & \cdots &  -\sum _{j=1}^{nh}w_{j}^{(2)}\tanh ^{(1)}(b_j)\sum _{k=1}^{n_y}v_{jk}^{(n)}e^{-i(\varOmega _1 +\varOmega _2)k\delta t}\\ \vdots &  \vdots &  \ddots &  \vdots \\ -\sum _{j=1}^{nh}w_{j}^{(n)}\tanh ^{(1)}(b_j)\sum _{k=1}^{n_y}v_{jk}^{(1)}e^{-i(\varOmega _1 +\varOmega _2)k\delta t} &  -\sum _{j=1}^{nh}w_{j}^{(n)}\tanh ^{(1)}(b_j)\sum _{k=1}^{n_y}v_{jk}^{(2)}e^{-i(\varOmega _1 +\varOmega _2)k\delta t} & \cdots &  1-\sum _{j=1}^{nh}w_{j}^{(n)}\tanh ^{(1)}(b_j)\sum _{k=1}^{n_y}v_{jk}^{(n)}e^{-i(\varOmega _1 +\varOmega _2)k\delta t} \end{bmatrix} \end{aligned} \nonumber \\&\quad \begin{aligned}&\begin{bmatrix} H_2^{(1:11)}(\varOmega _1,\varOmega _2) &  H_2^{(1:12)}(\varOmega _1,\varOmega _2) &  \cdots &  H_2^{(1:22)}(\varOmega _1,\varOmega _2) &  H_2^{(1:23)}(\varOmega _1,\varOmega _2) &  \cdots &  H_2^{(1:nn)}(\varOmega _1,\varOmega _2)\\ H_2^{(2:11)}(\varOmega _1,\varOmega _2) &  H_2^{(2:12)}(\varOmega _1,\varOmega _2) &  \cdots &  H_2^{(2:22)}(\varOmega _1,\varOmega _2) &  H_2^{(2:23)}(\varOmega _1,\varOmega _2) &  \cdots &  H_2^{(2:nn)}(\varOmega _1,\varOmega _2)\\ \vdots &  \vdots &  \vdots &  \vdots &  \vdots &  \ddots &  \vdots \\ H_2^{(n:11)}(\varOmega _1,\varOmega _2) &  H_2^{(n:12)}(\varOmega _1,\varOmega _2) &  \cdots &  H_2^{(n:22)}(\varOmega _1,\varOmega _2) &  H_2^{(n:23)}(\varOmega _1,\varOmega _2) &  \cdots &  H_2^{(n:nn)}(\varOmega _1,\varOmega _2) \end{bmatrix}\\&\quad = \begin{bmatrix} \alpha _2^{(1:11)} &  \alpha _2^{(1:12)} &  \cdots &  \alpha _2^{(1:22)} &  \alpha _2^{(1:23)} &  \cdots &  \alpha _2^{(1:nn)}\\ \alpha _2^{(2:11)} &  \alpha _2^{(2:12)} &  \cdots &  \alpha _2^{(2:22)} &  \alpha _2^{(2:23)} &  \cdots &  \alpha _2^{(2:nn)}\\ \vdots &  \vdots &  \vdots &  \vdots &  \vdots &  \ddots &  \vdots \\ \alpha _2^{(n:11)} &  \alpha _2^{(n:12)} &  \cdots &  \alpha _2^{(n:22)} &  \alpha _2^{(n:23)} &  \cdots &  \alpha _2^{(n:nn)} \end{bmatrix} \end{aligned} \end{aligned}$$

95 $$\begin{aligned}&\quad \begin{aligned} \alpha _2^{(p:ab)}&= \sum _{j=0}^{n_h}\frac{w_j^{(p)}\tanh ^{(2)}(b_j)}{2!}\Bigg (\sum _{m_1=0}^{n_x-1} \sum _{m_2=0}^{n_x-1}u_{jm_1}^{(a)}u_{jm_2}^{(b)}e^{-i\varOmega _1 m_1\delta t}e^{-i\varOmega _2 m_2\delta t} + \sum _{n_1=1}^{n}\Bigg (\sum _{m=0}^{n_x-1}\sum _{k=1}^{n_y}\Big (u_{jm}^{(b)}v_{jk}^{(n_1)} H_1^{(n_1:a)}(\varOmega _1)e^{-i\varOmega _1k\delta t}e^{-i\varOmega _2m\delta t} \\&+ u_{jm}^{(a)}v_{jk}^{(n_1)}H_1^{(n_1:b)}(\varOmega _2)e^{-i\varOmega _1m\delta t}e^{-i\varOmega _2k\delta t}\Big ) + \sum _{n_2=1}^{n}\Bigg (\sum _{k_1=1}^{n_y}\sum _{k_2=1}^{n_y}v_{jk_1}^{(n_1)}v_{jk_2}^{(n_2)} H_1^{(n_1:a)}(\varOmega _1)H_1^{(n_2:b)}(\varOmega _2)e^{-i\varOmega _1 k_1\delta t}e^{-i\varOmega _2 k_2\delta t}\Bigg )\Bigg )\Bigg ) \end{aligned} \end{aligned}$$

where *n* in relation to summations involving $n_1$ and $n_2$ is the number of degrees of freedom (input–output channels) available. The derivation also assumes that the kernel sizes for exogenous inputs and inputs ($n_x$ and $n_y$) are equivalent.

### $H_3$ kernel derivation (n orders)

The structure of the equation below follows logically from the recovery of $H_1$ and $H_2$. While the formulation of $\alpha$ becomes significantly more complex as the order increases, its derivation is relatively straightforward, albeit time-consuming. However, the primary advantage of recovering HFRFs in this manner is the flexibility in identifying nonlinearities in unknown systems from data.

96 $$\begin{aligned}&\begin{bmatrix} 1-\sum _{j=1}^{nh}w_{j}^{(1)}\tanh ^{(1)}(b_j)\sum _{k=1}^{n_y}v_{jk}^{(1)}e^{-i(\varOmega _1 +\varOmega _2 +\varOmega _3)k\delta t} &  -\sum _{j=1}^{nh}w_{j}^{(1)}\tanh ^{(1)}(b_j)\sum _{k=1}^{n_y}v_{jk}^{(2)}e^{-i(\varOmega _1 +\varOmega _2 +\varOmega _3)k\delta t} & \cdots &  -\sum _{j=1}^{nh}w_{j}^{(1)}\tanh ^{(1)}(b_j)\sum _{k=1}^{n_y}v_{jk}^{(n)}e^{-i(\varOmega _1 +\varOmega _2 +\varOmega _3)k\delta t}\\ -\sum _{j=1}^{nh}w_{j}^{(2)}\tanh ^{(1)}(b_j)\sum _{k=1}^{n_y}v_{jk}^{(1)}e^{-i(\varOmega _1 +\varOmega _2 +\varOmega _3)k\delta t} &  1-\sum _{j=1}^{nh}w_{j}^{(2)}\tanh ^{(1)}(b_j)\sum _{k=1}^{n_y}v_{jk}^{(2)}e^{-i(\varOmega _1 +\varOmega _2 +\varOmega _3)k\delta t} & \cdots &  -\sum _{j=1}^{nh}w_{j}^{(2)}\tanh ^{(1)}(b_j)\sum _{k=1}^{n_y}v_{jk}^{(n)}e^{-i(\varOmega _1 +\varOmega _2 +\varOmega _3)k\delta t}\\ \vdots &  \vdots &  \ddots &  \vdots \\ -\sum _{j=1}^{nh}w_{j}^{(n)}\tanh ^{(1)}(b_j)\sum _{k=1}^{n_y}v_{jk}^{(1)}e^{-i(\varOmega _1 +\varOmega _2 +\varOmega _3)k\delta t} &  -\sum _{j=1}^{nh}w_{j}^{(n)}\tanh ^{(1)}(b_j)\sum _{k=1}^{n_y}v_{jk}^{(2)}e^{-i(\varOmega _1 +\varOmega _2 +\varOmega _3)k\delta t} & \cdots & 1- \sum _{j=1}^{nh}w_{j}^{(n)}\tanh ^{(1)}(b_j)\sum _{k=1}^{n_y}v_{jk}^{(n)}e^{-i(\varOmega _1 +\varOmega _2 +\varOmega _3)k\delta t} \end{bmatrix} \nonumber \\&\begin{bmatrix} H_3^{(1:111)}(\varOmega _1,\varOmega _2,\varOmega _3) &  H_3^{(1:112)}(\varOmega _1,\varOmega _2,\varOmega _3) &  \cdots &  H_3^{(1:222)}(\varOmega _1,\varOmega _2,\varOmega _3) &  H_3^{(1:223)}(\varOmega _1,\varOmega _2,\varOmega _3) &  \cdots &  H_3^{(1:nnn)}(\varOmega _1,\varOmega _2,\varOmega _3)\\ H_3^{(2:111)}(\varOmega _1,\varOmega _2,\varOmega _3) &  H_3^{(2:112)}(\varOmega _1,\varOmega _2,\varOmega _3) &  \cdots &  H_3^{(2:222)}(\varOmega _1,\varOmega _2,\varOmega _3) &  H_3^{(2:223)}(\varOmega _1,\varOmega _2,\varOmega _3) &  \cdots &  H_3^{(2:nnn)}(\varOmega _1,\varOmega _2,\varOmega _3)\\ \vdots &  \vdots &  \vdots &  \vdots &  \vdots &  \ddots &  \vdots \\ H_3^{(n:111)}(\varOmega _1,\varOmega _2,\varOmega _3) &  H_3^{(n:112)}(\varOmega _1,\varOmega _2,\varOmega _3) &  \cdots &  H_3^{(n:222)}(\varOmega _1,\varOmega _2,\varOmega _3) &  H_3^{(n:223)}(\varOmega _1,\varOmega _2,\varOmega _3) &  \cdots &  H_3^{(n:nnn)}(\varOmega _1,\varOmega _2,\varOmega _3) \end{bmatrix}\nonumber \\&= \begin{bmatrix} \alpha _3^{(1:111)} &  \alpha _3^{(1:112)} &  \cdots &  \alpha _3^{(1:222)} &  \alpha _3^{(1:223)} &  \cdots &  \alpha _3^{(1:nnn)}\\ \alpha _3^{(2:111)} &  \alpha _3^{(2:112)} &  \cdots &  \alpha _3^{(2:222)} &  \alpha _3^{(2:223)} &  \cdots &  \alpha _3^{(2:nnn)}\\ \vdots &  \vdots &  \vdots &  \vdots &  \vdots &  \ddots &  \vdots \\ \alpha _3^{(n:111)} &  \alpha _3^{(n:112)} &  \cdots &  \alpha _3^{(n:222)} &  \alpha _3^{(n:223)} &  \cdots &  \alpha _3^{(n:nnn)} \end{bmatrix} \end{aligned}$$

97 $$\begin{aligned}&\alpha _3^{(p:abc)} =\sum _{j=0}^{n_h}\Bigg (\frac{2}{3}\frac{w_j^{(p)}\tanh ^{(2)}(b_j)}{2!} \Bigg (\sum _{n_1=1}^{n}\Bigg (\sum _{k=1}^{n_y}\sum _{m=0}^{n_x-1}\Big (v_{jk}^{(n_1)}u_{jm}^{(a)}H_2^{(n_1:bc)} (\varOmega _2,\varOmega _3)e^{-i\varOmega _1m\delta t}e^{-i(\varOmega _2 + \varOmega _3)k\delta t}\nonumber \\&\quad + v_{jk}^{(n_1)}u_{jm}^{(b)}H_2^{(n_1:ac)}(\varOmega _1,\varOmega _3)e^{-i\varOmega _2m\delta t}e^{-i(\varOmega _1 + \varOmega _3)k\delta t} + v_{jk}^{(n_1)}u_{jm}^{(c)}H_2^{(n_1:ab)}(\varOmega _1,\varOmega _2)e^{-i\varOmega _3m\delta t}e^{-i(\varOmega _1 + \varOmega _2)k\delta t}\Big ) \nonumber \\&\quad +\sum _{n_2=1}^{n}\Bigg (\sum _{k_1=1}^{n_y}\sum _{k_2=1}^{n_y}v_{jk_1}^{(n_1)}v_{jk_2}^{(n_2)}\Big (H_1^{(n_1:a)}(\varOmega _1) H_2^{(n_2:bc)}(\varOmega _2,\varOmega _3)e^{-i\varOmega _1k_1\delta t}e^{-i(\varOmega _2 + \varOmega _3)k_2\delta t}\nonumber \\&\quad + H_1^{(n_1:b)}(\varOmega _2)H_2^{(n_2:ac)}(\varOmega _1,\varOmega _3)e^{-i\varOmega _2k_1\delta t}e^{-i(\varOmega _1 + \varOmega _3)k_2\delta t}\nonumber \\&\quad + H_1^{(n_1:c)}(\varOmega _3)H_2^{(n_2:ab)}(\varOmega _1,\varOmega _2)e^{-i\varOmega _3k_1\delta t}e^{-i(\varOmega _1 + \varOmega _2)k_2\delta t}\Big )\Bigg )\Bigg )\Bigg ) + \frac{w_j^{(p)}\tanh ^{(3)}(b_j)}{3!}\nonumber \\&\quad \Bigg (\sum _{m_1=0}^{n_x-1}\sum _{m_2=0}^{n_x-1}\sum _{m_3=0}^{n_x-1}u_{jm_1}^{(a)}u_{jm_2}^{(b)}u_{jm_3}^{(c)}e^{-i\varOmega _1m_1\delta t}e^{-i\varOmega _2m_2\delta t}e^{-i\varOmega _3m_3\delta t}\nonumber \\&\quad + \sum _{n_1=1}^{n}\Bigg (\sum _{k_1=1}^{n_y}\sum _{m_1=0}^{n_x-1}\sum _{m_2=0}^{n_x-1}\Big (v_{jk_1}^{(n_1)}u_{jm_1}^{(a)}u_{jm_2}^{(b)} H_1^{(n_1:c)}(\varOmega _3)e^{-i\varOmega _1m_1\delta t}e^{-i\varOmega _2m_2\delta t}e^{-i\varOmega _3k_1\delta t} + v_{jk_1}^{(n_1)}u_{jm_1}^{(a)}u_{jm_2}^{(c)}H_1^{(n_1:b)}(\varOmega _2)e^{-i\varOmega _1m_1\delta t}e^{-i\varOmega _2k_1\delta t}e^{-i\varOmega _3m_2\delta t} \nonumber \\&\quad + v_{jk_1}^{(n_1)}u_{jm_1}^{(b)}u_{jm_2}^{(c)}H_1^{(n_1:a)}(\varOmega _1)e^{-i\varOmega _1k_1\delta t}e^{-i\varOmega _2m_1\delta t}e^{-i\varOmega _3m_2\delta t}\Big )\nonumber \\&\quad + \sum _{n_2=1}^{n}\Bigg (\sum _{k_1=1}^{n_y}\sum _{k_2=1}^{n_y}\sum _{m_1=0}^{n_x-1}\Big (v_{jk_1}^{(n_1)}v_{jk_2}^{(n_2)}u_{jm_1}^{(a)}H_1^{(n_1:b)}(\varOmega _2) H_1^{(n_2:c)}(\varOmega _3)e^{-i\varOmega _1m_1\delta t}e^{-i\varOmega _2k_1\delta t}e^{-i\varOmega _3k_2\delta t} \nonumber \\&\quad + v_{jk_1}^{(n_1)}v_{jk_2}^{(n_2)}u_{jm_1}^{(b)}H_1^{(n_1:a)}(\varOmega _1)H_1^{(n_2:c)}(\varOmega _3) e^{-i\varOmega _1k_1\delta t}e^{-i\varOmega _2m_1\delta t}e^{-i\varOmega _3k_2\delta t}\nonumber \\&\quad + v_{jk_1}^{(n_1)}v_{jk_2}^{(n_2)}u_{jm_1}^{(c)}H_1^{(n_1:a)}(\varOmega _1)H_1^{(n_2:b)}(\varOmega _2) e^{-i\varOmega _1k_1\delta t}e^{-i\varOmega _2k_2\delta t}e^{-i\varOmega _3m_1\delta t}\Big )\nonumber \\&\quad +\sum _{n_3=1}^{n}\Bigg (\sum _{k_1=1}^{n_y}\sum _{k_2=1}^{n_y}\sum _{k_3=1}^{n_y}v_{jk_1}^{(n_1)}v_{jk_2}^{(n_2)}v_{jk_3}^{(n_3)}H_1^{(n_1:a)} (\varOmega _1)H_1^{(n_2:b)}(\varOmega _2)H_1^{(n_3:c)}(\varOmega _3)e^{-i\varOmega _1k_1\delta t}e^{-i\varOmega _2k_2\delta t}e^{-i\varOmega _3k_3\delta t}\Bigg )\Bigg )\Bigg )\Bigg )\Bigg ) \end{aligned}$$

where *n* in relation to summations involving $n_1, n_2$ and $n_3$ is the number of degrees of freedom (input–output channels) available. The derivation also assumes that the lags for exogenous inputs and inputs ($n_x$ and $n_y$) are equal.
